# In Vitro Metabolism and Analytical Characterization of SLU‐PP‐332 and SLU‐PP‐915: Novel Pan‐ERR Agonists With Doping Potential

**DOI:** 10.1002/rcm.70039

**Published:** 2026-01-26

**Authors:** Tristan Möller, Oliver Krug, Mario Thevis

**Affiliations:** ^1^ Center for Preventive Doping Research/Institute of Biochemistry German Sport University Cologne Cologne Germany; ^2^ European Monitoring Center for Emerging Doping Agents (EuMoCEDA) Cologne/Bonn Germany

**Keywords:** in vitro metabolism, LC‐HRMS, metabolite synthesis, pan‐ERR agonists

## Abstract

**Rationale:**

Estrogen‐related receptor (ERR) agonists such as the drug candidates SLU‐PP‐332 and SLU‐PP‐915 are currently being investigated as exercise mimetics, given their ability to trigger human physiological processes similar to those initiated by actual physical activity. This capability prompted the consideration of these compounds as drugs potentially relevant for sports drug testing programs.

**Methods:**

The two pan‐ERR agonists SLU‐PP‐332 and SLU‐PP‐915 were characterized using liquid chromatography‐high resolution (tandem) mass spectrometry (LC–HRMS/MS). Furthermore, the in vitro metabolic transformation products of both compounds prepared by means of human liver S9 fraction (S9 fraction) and human liver microsomes (HLMs) were analyzed. In addition, selected metabolites of SLU‐PP‐915 were synthesized and their structures were analyzed by nuclear magnetic resonance (NMR) spectroscopy.

**Results:**

A total of nine metabolites were identified for SLU‐PP‐332, consisting of six Phase‐I metabolites and three Phase‐II conjugates. Conversely, the analysis of SLU‐PP‐915 yielded only Phase‐I transformation products, with a total of seven metabolites identified. In both cases, an in‐depth structural elucidation was conducted to obtain a comprehensive overview of the detected metabolites. Furthermore, three metabolites of SLU‐PP‐915 were confirmed through chemical synthesis and NMR.

**Conclusion:**

The results obtained in this study gave an in‐depth view into the analysis and in vitro metabolism of the newly developed pan‐ERR agonists SLU‐PP‐332 and SLU‐PP‐915. This may help to uncover the illicit use of these novel compounds as potential performance‐enhancing substances.

## 1 Introduction

The investigation of new physiological targets is a crucial aspect of drug research and development. One of these potential new targets is the class of estrogen‐related receptors (ERRs) [1]. These orphan receptors play an important role in energy homeostasis and lipid metabolism, and they have been associated with various diseases, such as metabolic or skeletal muscle disorders [2, 3, 4, 5]. Despite this receptor family's structural and sequence similarities to the estrogen receptors (ERs), they are not activated by their endogenous agonists [2, 6]. Therefore, potential ERR agonists have been investigated within recent years. Two of these newly developed agonists, SLU‐PP‐332 and SLU‐PP‐915, have been shown to target all three isoforms of the ERR (ERRα, ERRβ, and ERRγ), suggesting their potential for use in the treatment of various diseases [7]. Both compounds were investigated as pharmacological exercise mimetics, since these compounds were found to induce effects analogous to those of actual physical activity. SLU‐PP‐332 was demonstrated to promote an increase in type IIa oxidative skeletal muscle fibers and improve exercise endurance in animal models [8]. Similarly, SLU‐PP‐915 exhibited effects on gene expression and agonistic activity in vitro and in vivo [9]. These properties render them not only interesting as potential therapeutics to manage obesity or metabolic disorders, but also as potential substances of abuse in the context of sports. In order to maintain an effective anti‐doping system, it is vital that such compounds are analytically characterized prior to their market launch and, thus, availability to athletes. Non‐approved substances that exhibit the potential for performance enhancement can be subject of the World Anti‐Doping Agency (WADA) Prohibited List [10], and proactive analytical consideration in preventive anti‐doping research is critical.

When implementing new substances into existing doping control methods, it is essential to consider not only the intact compounds as target analytes but also their potential metabolites as these may allow for superior detection windows and improved sensitivity compared to approaches that exclusively consider the unmetabolized material [11]. In order to investigate the metabolic pathways of such compounds, in vitro metabolic approaches represent commonly employed first methods for the identification of potential metabolites. To date, limited data on the metabolism of SLU‐PP‐332 are available; for SLU‐PP‐915 no such studies are published [12]. Moreover, there is an absence of literature describing their mass spectrometric behavior, which would be of particular interest with regard to the evaluation of doping control urine samples.

This study presents the mass spectrometric characterization of SLU‐PP‐332 and SLU‐PP‐915. A thorough investigation was conducted into the in vitro metabolic behavior of both compounds. This investigation involved the identification and characterization of their potential metabolites. Furthermore, the chlorinated analog to SLU‐PP‐915 (referred to as SLU‐PP‐915‐Cl) was synthesized for its use as an internal standard (IS). In addition, the synthesis of selected metabolites of SLU‐PP‐915 was undertaken to validate their structures and to produce reference material for use in routine doping control analysis.

## 2 Methods

### 2.1 Chemicals and Reagents

SLU‐PP‐332 was purchased from Hycultec (Beutelsbach, Germany). 2‐chloroaniline, cesium carbonate, dimethyl formamide (DMF), 2‐fluoroaniline, hydrogen peroxide, magnesium chloride, *N*,*N*‐diisopropyl ethylamine (DIPEA), lithium hydroxide (LiOH), potassium dihydrogen phosphate, sodium hydroxide (NaOH), D‐saccharic acid‐1,4‐lactone (SL), and uridine diphosphate glucuronic acid (UDGPA) were obtained from Sigma Aldrich (St. Louis, MO, USA). 5‐bromothiophene‐2‐carboxylic acid, 1,4‐dioxane, human liver microsomes (HLMs), and human liver S9 fraction (S9 fraction) were obtained from Thermo Scientific (Bremen, Germany). Celite, magnesium sulfate (MgSO_4_), and tetrahydrofuran (THF) were purchased from Carl Roth (Karlsruhe, Germany). Ammonium acetate (AcNH_4_), dichloromethane (DCM), dimethyl sulfoxide (DMSO), TBTU, and tetrakis (triphenlyphosphine)palladium(0) ((PPh_3_)_4_Pd) were acquired from Merck (Darmstadt, Germany). Acetonitrile (ACN), acetic acid, cyclohexane, ethyl acetate (EtOAc), formic acid (FA), and *n‐pentane* were obtained from VWR Chemicals (Langenfeld, Germany). 1,3‐Phenyldiboronic acid was purchased from BLD Pharm (Reinbeck, Germany), and the nicotinamide adenine dinucleotide phosphate (NADPH) regenerating system was purchased from Promega (Madison, WIS, USA). MeOH was purchased from J.T.Baker (Phillipsburg, New Jersey, USA). Hydrogen gas (99.999%) was from Praxair (Düsseldorf, Germany). Ultrapure water was received from a Barnstead GenPure xCAD Plus from Thermo Scientific (Bremen, Germany).

Column chromatography was performed using silica gel (63–200 μm) from Supelco (Sigma Aldrich, St. Louis, Missouri, USA). For reaction control and control of the column chromatography, thin layer chromatography (TLC) plates were used from Merck (Darmstadt, Germany). Chromabond C18 6‐cc SPE cartridges were purchased from Macherey‐Nagel (Düren, Germany).

### 2.2 NMR Spectroscopy

For nuclear magnetic resonance (NMR) spectroscopy, a Bruker Avance I 300 and Bruker Avance III 499 system was used. ^1^H NMR‐spectra were acquired at a frequency of 300.1 or 499.9 MHz, while ^13^C NMR‐spectra were acquired at a frequency of 125.7 MHz. Peak assignments were facilitated by two‐dimensional spectra (H,H‐COSY, H,C‐HMBC, H,C‐HMQC). The chemical shift *σ* and the coupling constant 3*J* or 4*J* are indicated in parts per million and in hertz, respectively, and the multiplicity is classified as singlet (s), doublet (d), triplet (t), doublet doublet (dd), triplet triplet (tt), and multiplet (m). Spectra of all synthesized products are presented in the Supporting Information.

### 2.3 Synthesis

**FIGURE 1:**
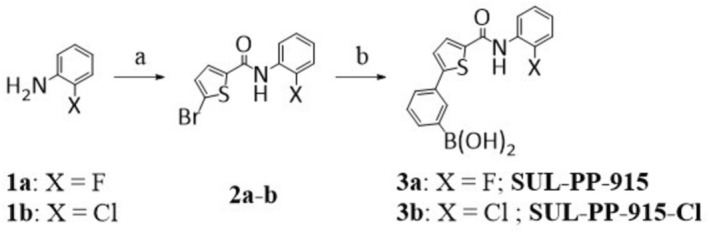
Synthesis of SLU‐PP‐915 and IS SLU‐PP‐915Cl. Reaction conditions: (a) aniline halide, TBTU, DiPEA, DMF, RT, overnight; (b) 1,3 phenyldiboronic acid, Cs_2_CO_3_, (PPh_3_)_4_Pd, DMF, H_2_O, 100°C, overnight.

General procedure for the amidation of the respective aniline halides (Figure 1a).

In a baked‐out flask, 5‐bromothiophene‐2‐carboxylic acid **1** (1 eq) was dissolved in dry DMF (1 mL/mmol) followed by the addition of TBTU (1.0 eq) and DiPEA (2.5 eq). After 20 min, the respective aniline halide (1.2 eq) was added, and the mixture was stirred overnight at room temperature (RT). The reaction was stopped by addition of H_2_O and was then extracted using EtOAc (3×). The combined organic phases were dried over MgSO_4_, and afterwards, the solvent was removed under reduced pressure. The crude product was then purified using column chromatography (*n*‐pentane: EtOAc = 5:1).

5‐bromo‐*N*‐(2‐fluorophenyl)thiophene‐2‐carboxamide **2a** was synthesized starting from 2‐fluoroaniline (0.64 g; 5.8 mmol) and was isolated as a gray solid with a yield of 0.89 g (61.8%).

^1^H NMR (DMSO‐d_6_; 500 MHz): *δ* 7.22 (m, 1H), 7.30 (m, 1H), 7.37 (d; *J* = 4.0 Hz, 1H), 7.58 (t; *J* = 7.7 Hz, 1H), 7.87 (d; *J* = 4.0 Hz, 1H), 10.25 (s, 1H).

^13^C NMR (DMSO‐d_6_; 125 MHz): *δ* 116.28 (C_quat__._), 118.50 (CH), 124.88 (C_quat__._), 125.34 (CH), 127.74 (C_quat__._), 130.80 (C_quat__._), 132.24 (C_quat__._), 141.736 (CH), 155.00 (CH), 157.45 (CH), 159.42 (CH).

5‐bromo‐*N*‐(2‐chlorophenyl)thiophene‐2‐carboxamide **2b** was synthesized starting from 2‐chloroaniline (0.30 g; 2.3 mmol) and was isolated as a yellowish oil with a yield of 0.44 g (72.0%).

^1^H NMR (DMSO‐d_6_; 500 MHz): *δ* 7.32 (t *J* = 7.7 Hz, 1H), 7.39 (m, 2H), 7.55 (m, 2H), 7.86 (d; *J* = 3.9 Hz, 1H), 10.21 (s, 1H).

^13^C NMR (DMSO‐d_6_; 125 MHz): *δ* 118.44 (CH), 128.04 (C_quat__._), 128.39 (C_quat__._), 129.28 (C_quat__._), 130.13 (C_quat__._), 130.23 (CH), 130.69 (C_quat__._), 132.25 (C_quat__._), 134.65 (CH), 141.35 (CH), 159.46 (CH).

General Procedure for the Suzuki Cross Coupling Reaction (Figure 1b)

The respective thiophene‐2‐carboamide (1.0 eq), 1,3 phenylenediboronic acid (1.3 eq), and Cs_2_CO_3_ (2.0 eq) were dissolved in 1,4‐dioxane (5 mL/mmol) and H_2_O (2 mL/mmol). Following the addition of (PPh_3_)_4_Pd (1 mol‐%), the reaction mixture stirred under reflux overnight. Afterwards, the reaction was cooled to RT and was then filtered over Celite. The solvent was removed under reduced pressure, and the crude product was washed with cyclohexane and DCM until the triphenylphosphine oxide residue vanished (screened via LC‐HRMS).

(3‐(5‐((2‐fluorophenyl)carbamoyl)thiophen‐2‐yl)phenyl)boronic acid **SLU‐PP‐915** was synthesized starting from **2a** (0.20 g; 0.7 mmol) and was found to form a grayish solid with a yield of 0.17 g (72.3%).

^1^H NMR (DMSO‐d_6_; 500 MHz): *δ* 7.22 (m, 2H), 7.30 (m, 3H), 7.6 (m, 3H), 7.76 (d; *J* = 8.0 Hz, 1H), 7.80 (d; *J* = 3.9 Hz, 1H), 8.09 (m, 2H), 10.24 (s, 1H).

^13^C NMR (DMSO‐d_6_; 125 MHz): *δ* 116.25 (CH), 116.44 (CH), 123.11 (CH), 124.80 (CH), 125.86 (CH), 126.05 (C_quat__._), 126.45 (CH), 127.39 (CH), 127.77 (CH), 130.83 (CH), 131.17 (CH), 134.44 (C_quat__._), 139.20 (C_quat__._), 147.94 (C_quat__._), 155.12 (C_quat__._), 157.57 (C_quat__._), 160.30 (C_quat__._).

(3‐(5‐((2‐chlorophenyl)carbamoyl)thiophen‐2‐yl)phenyl)boronic acid **SLU‐PP‐915‐Cl** was synthesized starting from **2b** (0.30 g; 1.0 mmol). It was isolated as a yellow solid with a yield of 0.23 g (64.7%).

^1^H NMR (DMSO‐d_6_; 500 MHz): *δ* 7.31 (m, 2H), 7.39 (m, 4H), 7.56 (m, 3H), 7.76 (d; *J* = 7.7 Hz, 1H), 7.88 (d; *J* = 3.9 Hz, 1H), 8.05 (s, 1H), 10.22 (s, 1H).

^13^C NMR (DMSO‐d_6_; 125 MHz): *δ* 118.48 (C_quat__._), 126.27 (CH), 127.81 (CH), 128.01 (CH), 128.33 (CH), 129.22 (CH), 129.74 (CH), 130.12 (CH), 130.22 (C_quat__._), 130.69 (CH), 132.22 (CH), 132.22 (CH), 134.58 (CH), 134.69 (C_quat__._), 138.43 (C_quat__._), 141.40 (C_quat__._), 149.09 (C_quat__._).

5‐(3‐Hydroxyphenyl)thiophene‐2‐Carboxylic Acid **M1**

**SLU‐PP‐915** (50 mg; 0.15 mmol; 1.0 eq) was dissolved in a solution of NaOH (6 M; 1 mL) and then H_2_O_2_ (30% in H_2_O; 1 mL) was added. The reaction mixture was stirred for 2 h at RT. Afterwards, LiOH (31 mg; 0.74 mmol; 5 eq) as well as THF (1.2 mL) and MeOH (0.2 mL) were added. The reaction was heated up to 70°C and was then stirred for 4 h. After cooling to RT, the reaction mixture was diluted with H_2_O and was then extracted with EtOAc (3×). The combined organic phases were dried over MgSO_4_ and afterwards the solvent was removed in vacuo. The crude product was then purified using a Chromabond C18 SPE cartridge (6 cc) eluting with ACN/H_2_O (30% to 80% ACN). After drying under a N_2_ stream the final product was isolated as a beige solid in quant. yield.

^1^H NMR (DMSO‐d_6_; 500 MHz): *δ* 6.80 (m, *J* = 7.9 Hz, 1H), 7.08 (s, 1H), 7.16 (d; *J* = 7.6 Hz, 1H), 7.26 (m, 1H), 7.45 (d; *J* = 3.6 Hz, 1H), 7.70 (d; *J* = 3.5 Hz, 1H).

^13^C NMR (DMSO‐d_6_; 125 MHz): *δ* 112.92 (C_quat__._), 116.50 (C_quat__._), 117.18 (C_quat__._), 124.84 (C_quat__._), 130.87 (C_quat__._), 133.51 (CH), 134.44 (CH), 134.73 (C_quat__._), 150.40 (CH), 158.42 (CH), 163.25 (CH).

5‐(3‐Boronophenyl)thiophene‐2‐Carboxylic Acid **M3**

To a solution of **SLU‐PP‐915** (50 mg; 0.15 mmol; 1.0 eq) dissolved in THF (1.2 mL), MeOH (0.2 mL) and H_2_O (0.6 mL), LiOH (62 mg; 1.47 mmol; 10 eq) was added and the mixture was stirred at 70°C for 6 h. Afterwards, the reaction was cooled down to RT and was then extracted with EtOAc (3×). The combined organic phases were dried using MgSO_4_ and the solvent was removed under reduced pressure. The residue was further purified using a Chromabond C18 SPE cartridge (6 cc) eluting with ACN/H_2_O (30% to 80% ACN). Finally, the product was isolated after drying under a N_2_ stream and was found to produce a beige solid in quant. yield.

^1^H NMR (D_2_O; 500 MHz): *δ* 7.48 (m, 3H), 7.58 (d; *J* = 7.2 Hz, 1H), 7.84 (s, 1H), 8.38 (s, 1H).

^13^C NMR (D_2_O; 125 MHz): *δ* 123.32 (CH), 124.50 (CH), 128.10 (CH), 128.73 (CH), 131.92 (CH), 132.07 (CH), 134.35 (C_quat__._), 139.31 (C_quat__._), 132.24 (C_quat__._), 147.37 (C_quat__._), 149.82 (C_quat__._).

*N*‐(2‐Fluorophenyl)‐5‐(3‐Hydroxyphenyl)thiophene‐2‐Carboxamide **M4**

**SLU‐PP‐915** (50 mg; 0.15 mmol; 1.0 eq) was dissolved in a solution of NaOH (6 M; 1 mL) and H_2_O_2_ (30% in H_2_O; 1 mL). Following, the reaction mixture was stirred for 2 h at RT. Without further purification, the reaction mixture was loaded onto a Chromabond C18 SPE cartridge (6 cc) and eluted with ACN/H_2_O (0% to 20% ACN). The solvent was removed using an N_2_ stream to afford the final product as a brown solid in quant. yield.

^1^H NMR (DMSO‐d_6_; 500 MHz): δ 6.79 (d, *J* = 8.0 Hz, 1H), 7.13 (m, 2H), 7.27 (m, 5H), 7.52 (d; *J* = 3.8 Hz, 1H), 7.60 (t, *J* = 7.6 Hz, 1H), 7.98 (d; *J* = 3.6 Hz, 1H), 10.14 (s, 1H).

^13^C NMR (DMSO‐d_6_; 125 MHz): δ 113.43 (CH), 115.67 (CH), 116.28 (CH), 116.47 (CH), 117.32 (CH), 124.54 (CH), 124.98 (CH), 125.22 (C_quat__._), 127.57 (CH), 130.87 (CH), 131.36 (CH), 134.23 (C_quat__._), 137.06 (C_quat__._), 149.96 (C_quat__._), 155.04 (C_quat__._), 157.51 (C_quat__._), 160.85 (C_quat__._).

### 2.4 In Vitro Metabolic Assay

In vitro incubation experiments were conducted using a protocol slightly deviating from the protocol described by Kuuranne et al. [13]. HLMs and S9 fraction were used to perform both Phase‐I and Phase‐II metabolism and for NADPH supply a NADPH regenerating system (NADPH reg system) was used. SLU‐PP‐332 was diluted in MeOH, while SLU‐PP‐915 and SLU‐PP‐915‐Cl were diluted in DMSO to produce stock solutions with concentrations of 1 and 2 mg/mL, respectively. In order to obtain suitable working solution with a concentration of 200 μM, the stock solutions were diluted in 50 mM phosphate buffer (pH 7.4) containing 5 mM MgCl_2_. All incubation experiments were conducted in triplicate, using 10 μL working solution, 10 μL NADPH regenerating system (50 mM), 5 μL HLM (20 mg/mL), and 5 μL S9 fraction (20 mg/mL) for Phase‐I incubation experiments. After addition of 20 μL phosphate buffer for a total volume of 50 μL, all samples were incubated at 37°C for 24 h.

For Phase‐II metabolism, additional 5 μL of HLM and S9 fraction, as well as 10 μL of UDGPA (50 mM), 10 μL SL (50 mM), and 10 μL PAPS (20 μM) were added and the mixture was again incubated at 37°C for 24 h. To verify the results obtained in this study, blank samples either excluding enzymes (enzyme blank) or excluding substrate (substrate blank) were prepared. These samples were also used to identify metabolites obtained from non‐enzymatic transformations. Metabolic reactions were stopped by the addition of 150 μL ice‐cold ACN. The supernatant was harvested after centrifugation (17 000 × *g*, 5 min) and transferred into a fresh tube. After drying using a vacuum centrifuge (45°C, 45 min), the samples were reconstituted in 100 μLH_2_O:ACN (90:10 *v/v*).

### 2.5 Liquid Chromatography‐High Resolution (Tandem) Mass Spectrometry (LC–HRMS/MS)

LC‐HRMS/MS measurements were performed using a Vanquish UHPLC system coupled to an Orbitrap Exploris 480 mass spectrometer both manufactured by Thermo Fisher (Bremen, Germany). The HPLC system was equipped with an EC 4/3 Nucleoshell RP 18 Plus guard column (4 × 3 mm, 5‐μm particle size) from Macherey–Nagel (Düren, Germany) and a Poroshell 120 EC C18 column (3.0 × 50 mm, 2.7 μm) by Agilent (Santa Clara, California, USA). The chromatographic conditions were optimized for the different compounds analyzed in this study, using 50 mM AcNH_4_‐buffer as eluent A in the case of SLU‐PP‐332 and 0.1% FA in H_2_O in the case of SLU‐PP‐915 and SLU‐PP‐915‐Cl. In any case, 0.1% FA in ACN was used as eluent B. The gradient elution with each solvent system started at 0% B and was then increased to 100% B within 10 min, where it was held for 2 min. After returning to starting conditions within 0.01 min, the column was re‐equilibrated for 2 min. A flow of 0.3 mL/min was applied. The injection volume was 10 μL.

The HRMS parameters were optimized to obtain the best possible results for each analyte. A heated electrospray ionization (ESI) source was used that was either run in positive or negative ionization mode with a voltage of 3000 or −2600 V, respectively. Both full scan data and product ion scans were monitored. In full scan mode, the system operated at a resolution of 60 000 full width at half maximum (FWHM) and a scan range of *m*/*z* 80–800. Product ion scans were generated using parallel reaction monitoring (PRM) at a resolution of 30 000 FWHM. The isolation window was set to *m*/*z* 1.3. For higher energy collisional dissociation (HCD) in positive and negative ionization mode, normalized collision energies of 40% were used. In addition, pseudo MS^3^ experiments were conducted, using in‐source fragmentation. Nitrogen was used as collision gas and was generated by a CMC nitrogen generator (Eschborn, Germany). The HRMS was regularly calibrated using the Pierce Flex Mix calibration solution from Thermo Fisher (Bremen, Germany).

## 3 Results and Discussion

### 3.1 Synthesis of Reference Material and Mass Spectrometric Characterization

The mass spectrometric characterization is a crucial part of the implementation of new compounds into existing doping control methods or to establish novel methods for those substances. SLU‐PP‐332 has been shown to be detectable in both positive and negative ionization modes. However, with regard to the results obtained for the in vitro metabolic transformations, positive ionization was selected for further analysis.

**FIGURE 2:**
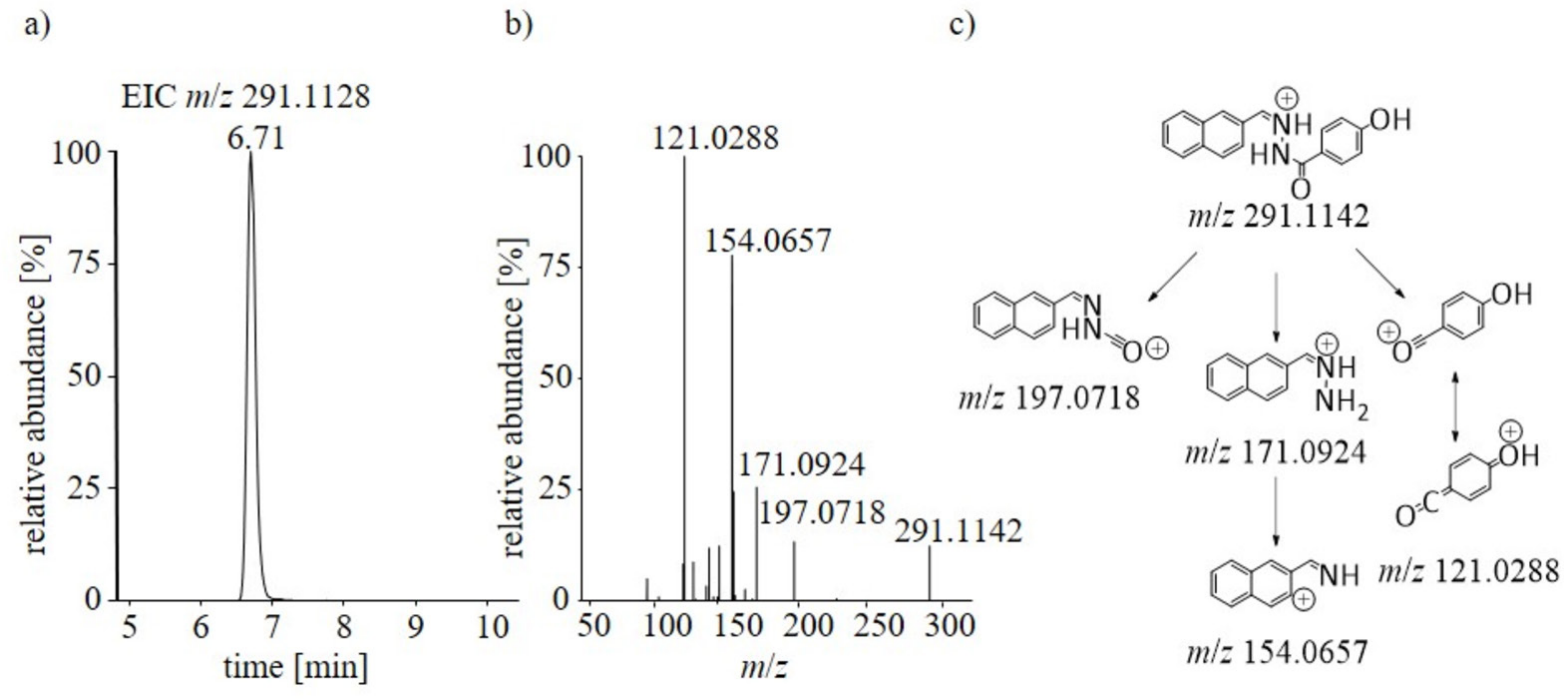
(a) Extracted ion chromatogram (EIC) of SLU‐PP‐332; (b) MS^2^ spectrum obtained for SLU‐PP‐332; (c) proposed dissociation pathway of SLU‐PP‐332. Measurements were conducted in positive ionization mode.

In positive ionization mode, SLU‐PP‐332 was detectable as [M+H]^+^ (*m*/*z* 291.1142). Evaluating MS^2^ data, *m*/*z* 121.0288 was identified as the most abundant peak and is most likely produced by *α*‐cleavage of the hydrazide group. Interestingly, the counterpart at *m*/*z* 171.0924 was also detected. Probably, this ion is produced by transferring a proton from the ion at *m*/*z* 121.0288 by forming a ion/neutral complex as described in the literature [14, 15]. This product ion is suggested to further eliminate ammonia (17 u) to produce a signal at *m*/*z* 154.0657. Another *α*‐cleavage, most likely occurring at the keto group, yielded the product ion at *m*/*z* 197.0718. The results obtained for SLU‐PP‐332 are summarized in Figure 2.

Due to the limited availability of SLU‐PP‐915 reference material, it was synthesized in‐house using a synthesis approach previously described in the literature [9]. In brief, 2‐fluoroaniline **1a** was reacted with 5‐bromothiophene‐2‐carboxylic acid to produce intermediate **2a**. Subsequently, SLU‐PP‐915 was formed by transforming **2a** using a Suzuki cross coupling reaction. The IS was synthesized analogously, starting from the chloro analog **1b**. Both reaction procedures are outlined in Figure 1.

**FIGURE 3:**
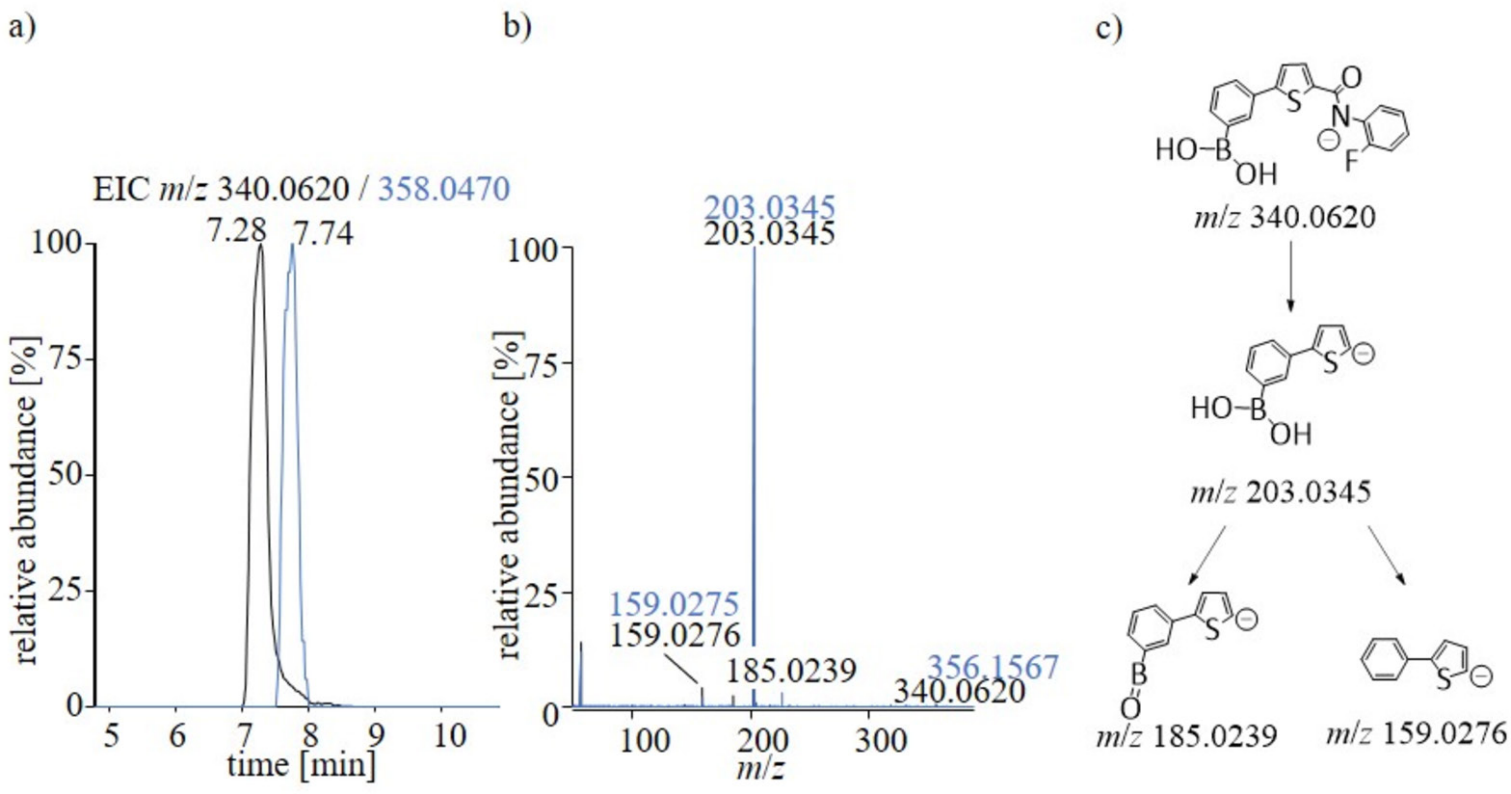
(a) EIC for SLU‐PP‐915 (black) and SLU‐PP‐915‐Cl (blue); (b) MS^2^ spectrum obtained for SLU‐PP‐915 (black) and SLU‐PP‐Cl (blue); (c) proposed dissociation pathway of SLU‐PP‐915. Measurements were conducted in negative ionization mode.

For SLU‐PP‐915 and the IS, the negative ionization mode was chosen for characterization and further analysis of its in vitro metabolic behavior. As shown in Figure 3, both compounds display comparable chromatographic properties and a similar dissociation pattern. Consequently, the mass spectrometric characterization is addressed at unison. Both compounds produce a major product ion at *m*/*z* 203.0345 which is most probably attributed to the α‐cleavage at the keto function. Pseudo‐MS^3^ experiments further indicate that this product ion undergoes further dissociation by cleaving either H_2_O (18 u) or by releasing its boronic acid function (44 u) to produce the product ions at *m*/*z* 185.0239 and *m*/*z* 159.0276, respectively.

### 3.2 In Vitro Metabolic Transformation

Within this study, the in vitro metabolic experiments were conducted using established methods involving the usage of HLMs and S9 fraction; further cofactors were added to promote Phase‐II metabolism [13]. However, the in vitro metabolic pathways of SLU‐PP‐332 were already described [12]. This study aimed to provide new insights and further information on the in vitro metabolism of this substance. The metabolic transformations cover a wide range of reactions, including hydroxylation (**M1a‐b**), bishydroxylation (**M2a‐b**), bishydroxylation, and reduction (**M3a‐b**), as well as Phase‐II transformations such as glucuronidation (**M4**), sulfation (**M5**), and the subsequent hydroxylation followed by sulfation (**M6**). In the literature, similar transformations such as hydroxylation, bishydroxylation, combined bishydroxylation and reduction, and glucuronidation were described. However, hydroxylation with subsequent glucuronidation was described beforehand but could not be identified in this study. In contrast to the literature, within this study the formation of different sulfates were accomplished. An overview of the transformation products of SLU‐PP‐332 observed in this study can be found in Table 1.

**TABLE 1:** List of the metabolic transformations identified for SLU‐PP‐332, including the product ions obtained after MS^2^ analysis. For identification, signals within a maximum mass error of 5 ppm were accepted. MS^2^ experiments were conducted using a normalized collision energy of 40%. For each metabolite the most abundant signal is highlighted in bold.

compound	Metabolic transformation	[M + H]^+^ (theo.) (*m*/*z*)	Formula	RT (min)	Product ions (exp.)	Proposed formula
SLU‐PP‐332		291.1128	C_18_H_15_N_2_O_2_^+^	6.71	197.0718	C_12_H_9_N_2_O^+^
					171.0924	C_11_H_11_N_2_^+^
					154.0657	C_11_H_8_N^+^
					**121.0288**	**C**_**7**_ **H**_**5**_ **O**_**2**_^**+**^
**332‐M1a**	Hydroxylation	307.1077	C_18_H_15_N_2_O_3_^+^	4.43	187.0874	C_11_H_11_N_2_O^+^
					170.0606	C_11_H_8_NO^+^
					138.0554	C_7_H_8_NO_2_^+^
					**121.0288**	**C**_**7**_ **H**_**5**_ **O**_**2**_^**+**^
**332‐M1b**	Hydroxylation	307.1077	C_18_H_15_N_2_O_3_^+^	6.36	197.0717	C_12_H_9_N_2_O^+^
					171.0924	C_11_H_11_N_2_^+^
					154.0658	C_11_H_8_N^+^
					**137.0238**	**C**_**7**_ **H**_**5**_ **O**_**3**_^**+**^
					111.0444	C_6_H_7_O_2_^+^
**332‐M2a**	Bishydroxylation	323.1026	C_18_H_15_N_2_O_4_^+^	5.17	**280.0978**	**C**_**17**_ **H**_**14**_ **NO**_**3**_^**+**^
					203.0821	C_11_H_11_N_2_O_2_^+^
					262.0872	C_17_H_12_NO_2_^+^
					121.0289	C_7_H_5_O_2_^+^
**332‐M2b**	Bishydro‐xylation	323.1026	C_18_H_15_N_2_O_4_^+^	5.41	**280.0978**	**C**_**17**_ **H**_**14**_ **NO**_**3**_^**+**^
					262.0875	C_17_H_12_NO_2_^+^
					121.0290	C_7_H_5_O_2_^+^
**332‐M3a**	Reduction + Bishydro‐xylation	325.1183	C_18_H_17_N_2_O_4_^+^	4.41	307.1089	C_18_H_15_N_2_O_3_^+^
					188.0715	C_11_H_10_NO_2_^+^
					170.0606	C_11_H_8_NO^+^
					161.0603	C_10_H_9_O_2_^+^
					**121.0288**	**C**_**7**_ **H**_**5**_ **O**_**2**_^**+**^
**332‐M3b**	Reduction + Bishydro‐xylation	325.1183	C_18_H_17_N_2_O_4_^+^	4.64	188.0717	C_11_H_10_NO_2_^+^
					170.0608	C_11_H_8_NO^+^
					161.0603	C_10_H_9_O_2_^+^
					**121.0289**	**C**_**7**_ **H**_**5**_ **O**_**2**_^**+**^
**332‐M4**	Glucuronidation	467.1449	C_24_H_23_N_2_O_8_^+^	5.79	**291.1141**	**C**_**18**_ **H**_**15**_ **N**_**2**_ **O**_**2**_^**+**^
					197.0718	C_12_H_9_N_2_O^+^
					171.0924	C_11_H_11_N_2_^+^
					154.0658	C_11_H_8_N^+^
					121.0289	C_7_H_5_O_2_^+^
**332‐M5**	Sulfation	371.0696	C_18_H_15_N_2_O_5_S^+^	6.51	291.1141	C_18_H_15_N_2_O_2_^+^
					**154.0658**	**C**_**11**_ **H**_**8**_ **N** ^**+**^
					121.0287	C_7_H_5_O_2_^+^
**332‐M6**	Hydroxylation + Sulfation	387.0645	C_18_H_15_N_2_O_6_S^+^	6.61	307.1095	C_18_H_15_N_2_O_3_^+^
					197.0718	C_12_H_9_N_2_O^+^
					171.0924	C_11_H_11_N_2_^+^
					**154.0658**	**C**_**11**_ **H**_**8**_ **N** ^**+**^
					137.0239	C_7_H_5_O_3_^+^

**FIGURE 4:**
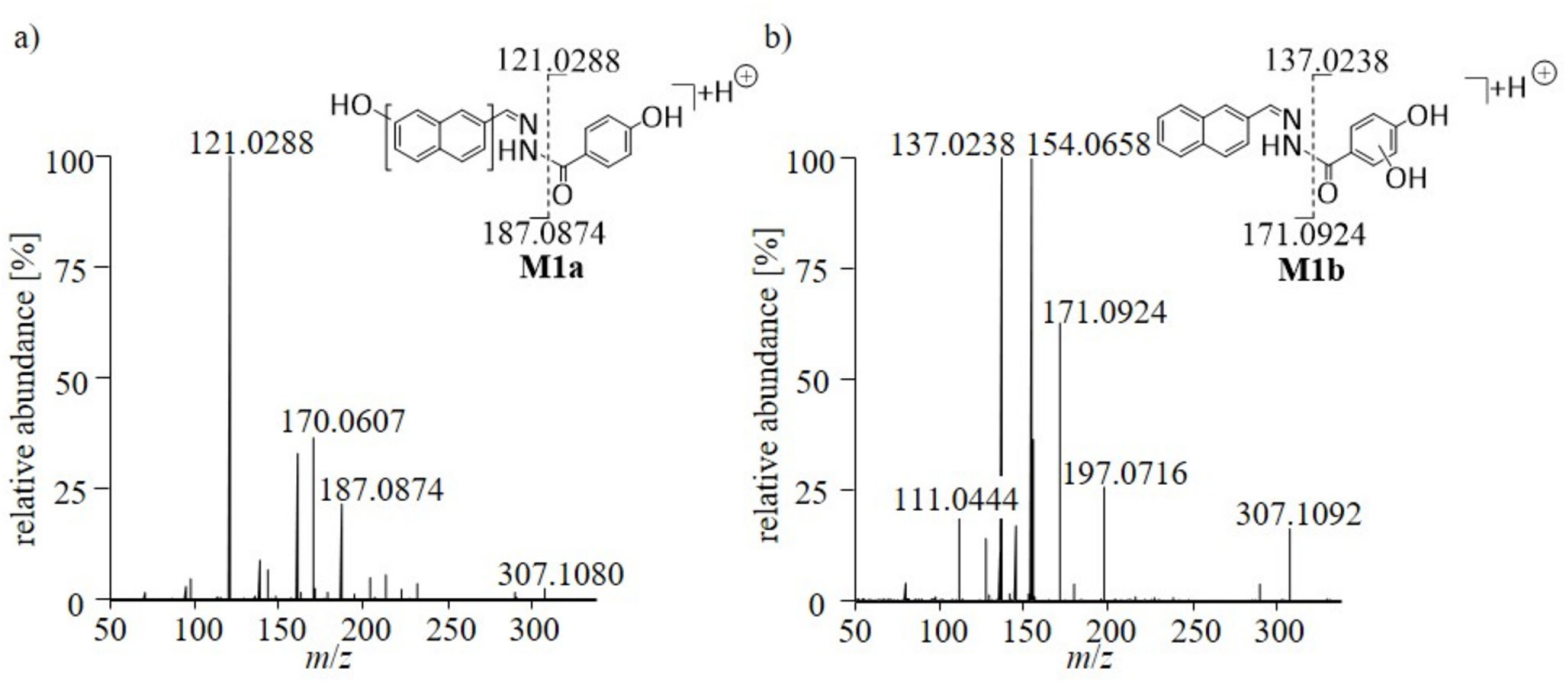
MS^2^ spectra and proposed structures of the hydroxylated metabolites (a) **M1a** and (b) **M1b** of SLU‐PP‐332.

For structural elucidation, the MS^2^ data were further evaluated. As exemplified for metabolite group **M1** (hydroxylation; *m*/*z* 307.1077) the extracted product ion spectra were used to determine the position of the hydroxy groups within the molecules. For metabolite **M1a**, the product ions at *m*/*z* 121.0288 and 187.0874 most likely result from α‐cleavage of the amide bond, thus indicating hydroxylation on the naphthalene function of the molecule (product ion *m*/*z* 187.0874 shown in Figure 1, left). In addition, the product ions *m*/*z* 138.0554 and 170.0607, proposed to result from cleavage of the hydrazine group (not shown), further corroborate this assumption. For metabolite **M1b**, similar results were obtained, showing again α‐cleavage of the amide bond to produce the signals at *m*/*z* 137.0238 and *m*/*z* 171.0924, indicating hydroxylation on the phenolic function of the molecule. Moreover, additional product ions at *m*/*z* 111.0444 and 197.0715, probably resulting from *α*‐cleavage of the benzaldehyde bond (not shown), substantiate this interpretation. The MS^2^ spectra and the proposed structure of **M1a** and **M1b** can be found in Figure 4. The metabolites **M2a‐b** (bishydroxylation) were analyzed likewise. In both cases, the MS^2^ spectra indicate both transformations on the naphthalene ring of the metabolites.

**FIGURE 5:**
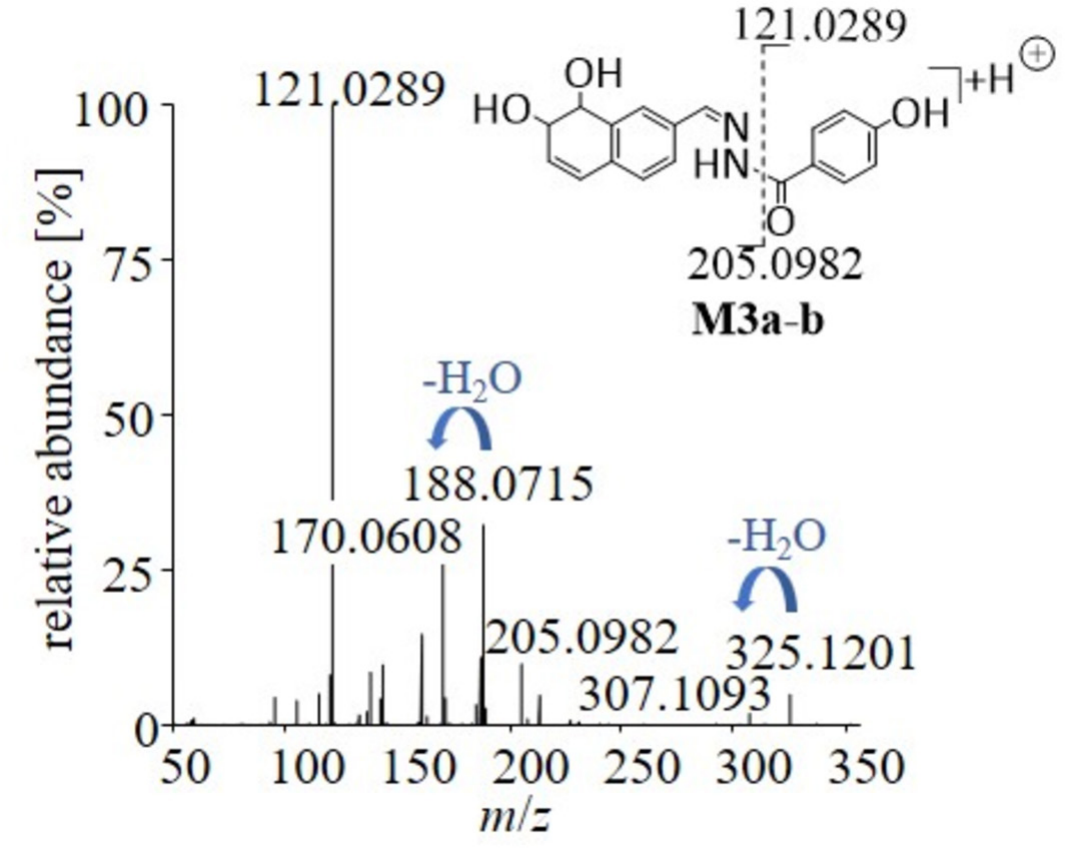
MS^2^ spectra and proposed structure of the reduced and bishydroxylated metabolites M3a‐b of SLU‐PP‐332. Losses of H_2_O are indicated with blue arrows.

The metabolite group **M3a‐b** presumably results from bishydroxylation together with a reductive transformation and is already mentioned in the literature [12]. However, due to its unusual nature, this transformation was further investigated within this study. Reviewing the MS^2^ spectra, the metabolization can be located on the naphthalene ring within the molecules. Further, the MS^2^ data support the assignment of losses of water (−18 u), which would contribute to re‐establish the metabolically interrupted aromatic system, supporting the proposed structure of the metabolites shown in Figure 5. As described in the literature, naphthalene itself is known to be transformed in vivo and in vitro to produce its corresponding epoxide, naphthalene oxide, which is then further transformed into the metabolite naphthalene dihydrodiol [16, 17]. A similar transformation pattern may thus be assumed for SLU‐PP‐332 as described here.

**FIGURE 6:**
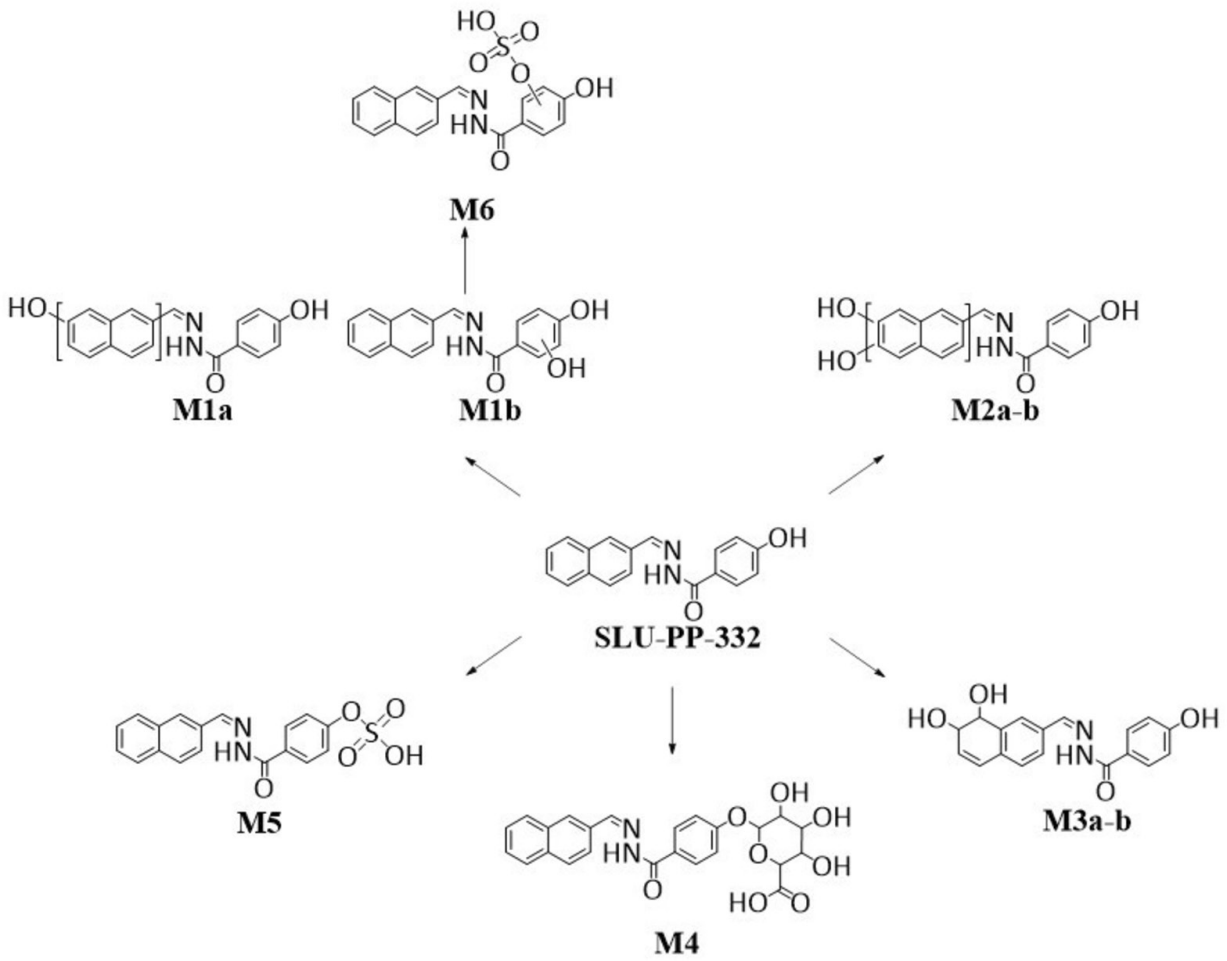
Overview of the metabolic pattern of SLU‐PP‐332.

Besides phase‐I transformation products, also Phase‐II conjugates were identified. Both glucuronidation and sulfation of the parent compound were observed, yielding the metabolites **M4** (*m*/*z* 467.1449) and **M5** (*m*/*z* 371.0696), respectively. In addition, SLU‐PP332 underwent hydroxylation and subsequent sulfation to form a metabolite at *m*/*z* 387.0645 (**M6**). The dissociation pattern of **M6**, however, gave similar results to those obtained for metabolite **M1b** (see Table 1), therefore, **M6** can be tentatively assigned to the sulfate conjugate of metabolite **M1b**. Altogether, a total of nine metabolites comprising six Phase‐I metabolites and three phase‐II conjugates were identified for SLU‐PP‐332. An overview of the metabolic pattern of SLU‐PP 332 is shown in Figure 6.

The in vitro metabolic behavior of SLU‐PP‐915 was investigated as described for SLU‐PP‐332. However, for this compound only phase‐I transformation products were identified. The metabolic reactions ranged from amide hydrolysis, oxidation of the boronic acid, and hydroxylation to form the metabolites **M1** (amide hydrolysis and oxidation), **M2** (amide hydrolysis, oxidation, and hydroxylation), **M3** (amide hydrolysis), **M4** (oxidation), **M5a‐b** (oxidation and hydroxylation), and **M6** (hydroxylation). Remarkably, oxidation to convert the boronic acid moiety into a hydroxy‐group, as observed for the metabolites **M4** and **M5**, is frequently described in the literature [18, 19]. However, it is important to mention that metabolites **M1**, **M3**, and **M4** were also detectable in the enzyme blank, indicating a non‐enzymatic metabolic pathway. Nevertheless, these metabolites could also be formed in vivo and might thus be no less interesting as putative targets for doping control analysis. An overview of the transformation products of SLU‐PP‐915 observed in this study can be found in Table 2.

**TABLE 2:** List of the metabolic transformations identified for SLU‐PP‐915, including the product ions obtained after MS^2^ analysis. For identification, signals within a maximum mass error of 5 ppm were accepted. MS^2^ experiments were conducted using a collision energy of 40%. For each metabolite, the most abundant signal is highlighted in bold.

Compound	Metabolic transformation	[M‐H]^−^ (theo.) (*m*/*z*)	Formula	RT (min)	Product ions (exp.)	Proposed formula
SLU‐PP‐915		340.0620	C_17_H_12_O_3_NBFS^−^	7.11	**203.0340**	**C**_**10**_ **H**_**8**_ **O**_**2**_ **BS** ^**−**^
					185.0249	C_10_H_6_BOS^−^
					159.0284	C_10_H_7_S^−^
SLU‐PP‐915‐Cl (IS)		356.0325	C_17_H_12_O_3_NBClS^−^	7.76	**203.0356**	**C**_**10**_ **H**_**8**_ **O**_**2**_ **BS** ^**−**^
					185.0248	C_10_H_6_BOS^−^
					159.0284	C_10_H_7_S^−^
**915‐M1**	Amide hydrolysis + oxidation	219.0121	C_11_H_7_O_3_S^−^	5.97	**175.0220**	**C**_**10**_ **H**_**7**_ **OS** ^**−**^
**915‐M2**	Amide hydrolysis + oxidation + Hydroxylation	235.0071	C_11_H_7_O_4_S^−^	5.19	**191.0170**	**C**_**10**_ **H**_**7**_ **O**_**2**_ **S** ^**−**^
**915‐M3**	Amide hydrolysis	247.0242	C_11_H_8_BO_4_S^−^	5.56	**203.0341**	**C**_**10**_ **H**_**8**_ **O**_**2**_ **BS** ^**−**^
					159.0272	C_10_H_7_S^−^
**915‐M4**	Oxidation	312.0500	C_17_H_11_FNO_2_S^−^	7.55	292.0436	C_17_H_10_NO_2_S^−^
					**174.0220**	**C**_**10**_ **H**_**7**_ **OS** ^**−**^
**915‐M5a**	Oxidation + hydroxylation	328.0449	C_17_H_11_FNO_3_S^−^	6.49	**308.0385**	**C**_**17**_ **H**_**10**_ **NO**_**3**_ **S** ^**−**^
					152.0151	C_7_H_3_FNO_2_^−^
					175.0232	C_10_H_7_OS^−^
**915‐M5b**	Oxidation + hydroxylation	328.0449	C_17_H_11_FNO_3_S^−^	6.83	308.0385	C_17_H_10_NO_3_S^−^
					**191.0181**	**C**_**10**_ **H**_**7**_ **O**_**2**_ **S** ^**−**^
**915‐M6**	Hydroxylation	356.0570	C_17_H_12_BFNO_4_S^−^	6.16	**338.0490**	**C**_**17**_ **H**_**10**_ **BFNO**_**3**_ **S** ^**−**^
					318.0424	C_17_H_9_BNO_3_S^−^
					152.0163	C_7_H_3_FNO_2_^−^

**FIGURE 7:**
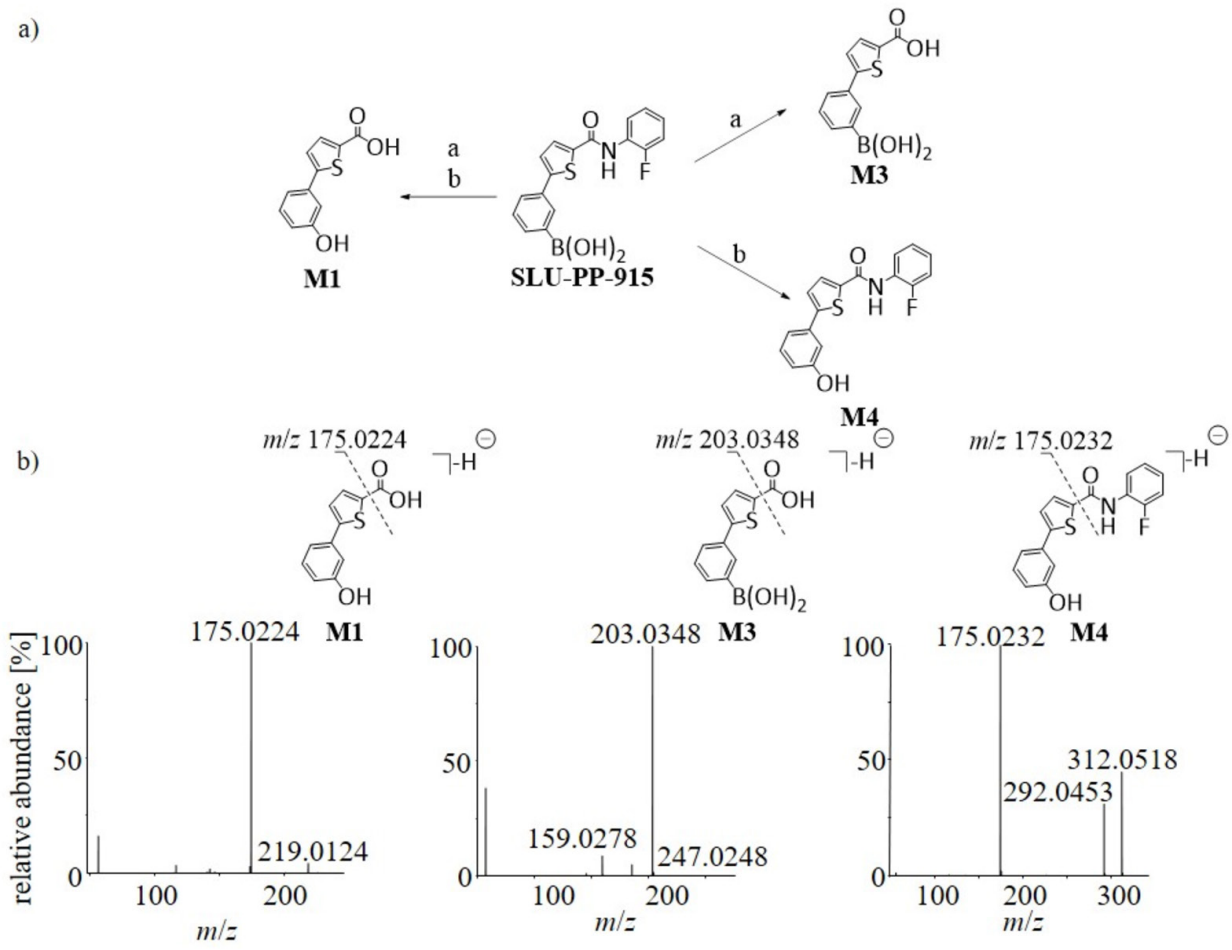
a) Synthesis of the metabolites **M1**, **M3** and **M4**. Reaction conditions: (a) NaOH, H_2_O_2_, H_2_O, RT, 2 h; (b) LiOH, THF, MeOH H_2_O, 70°C, 4–6 h. b) MS^2^ spectra obtained for the metabolites **M1**, **M3**, and **M4** of SLU‐PP‐915.

For structural conformation and to provide reference material for routine doping control testing, the metabolites **M1**, **M3**, and **M4** were synthesized using the reaction conditions shown in Figure 7a. In each case, the chromatographic and mass spectrometric properties of the reference material matched those of the in vitro generated metabolites, confirming the proposed structures. Upon collision‐induced dissociation, all metabolites showed α‐cleavage yielding the most abundant signal within the MS^2^ spectra at *m*/*z* 175.0224 for **M1**, *m*/*z* 203.0348 for **M3**, and *m*/*z* 175.0232 for **M4**. Further, **M3** showed a second product ion at *m*/*z* 159.0278, which can most likely be assigned to the chemical formula of C_10_H_7_S^−^. **M4**, however, showed the additional elimination of HF (20 u), generating a product ion at *m*/*z* 292.0453. The MS^2^ spectra of each metabolite can be found in Figure 7b.

**FIGURE 8:**
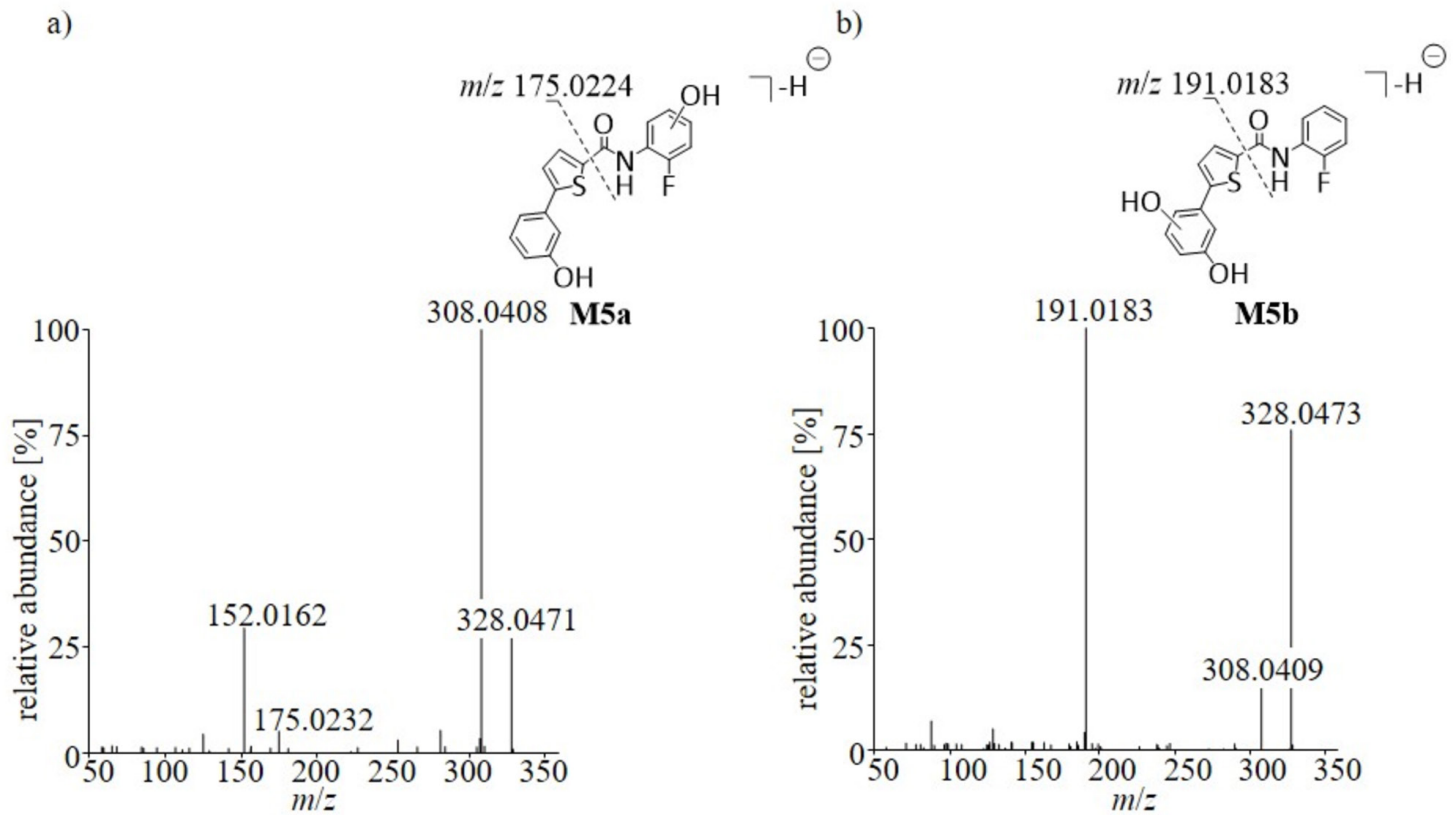
MS^2^ spectra and proposed structures of the oxidized and hydroxylated metabolites (a) **M5a** and (b) **M5b** of SLU‐PP‐915.

The MS^2^ spectra of the remaining metabolites were further analyzed, to obtain information on the structures of the produced metabolites. As indicated in Table 2, the metabolite group **M5** (oxidation and hydroxylation) included two metabolites with varying product ions, indicating different positions of the OH‐groups. As described for the synthesized metabolites **M1**, **M3** and **M4**, both compounds showed *α*‐cleavage resulting in the product ions at *m*/*z* 175.0232 for **M5a** and *m*/*z* 191.0183 for **M5b**, that are most likely assigned to the 3‐(thiophen‐2‐yl)phenol ion and its hydroxylated counterpart, respectively. In case of **M5a** this interpretation can be further underlined by the product ion at *m*/*z* 152.0162 which can be attributed to the hydroxylated aniline ring within the molecule. Based on this data, the structures indicated in Figure 8 were proposed.

**FIGURE 9:**
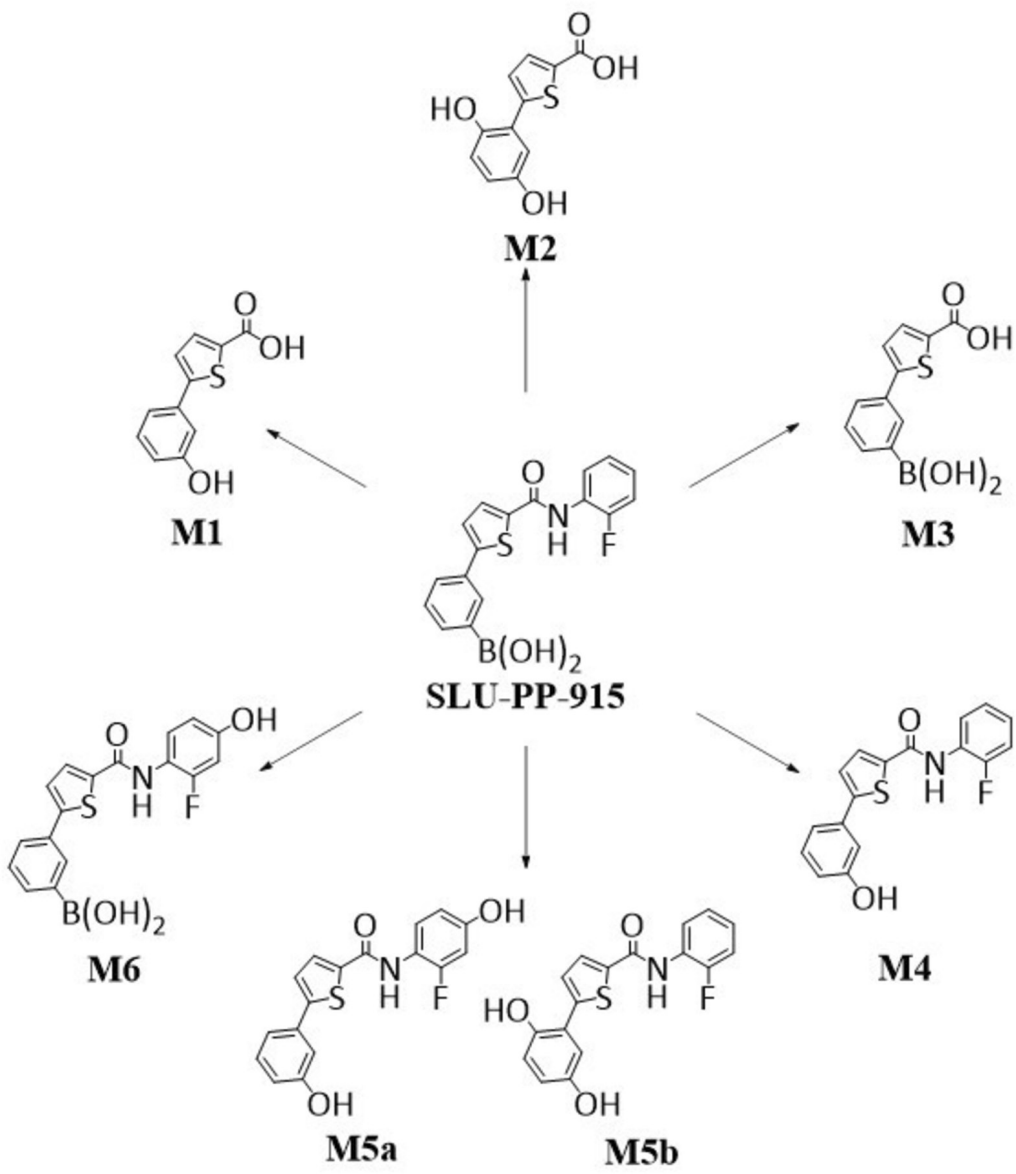
Overview of the metabolic pattern of SLU‐PP‐915.

As described for **M5a‐b**, the MS^2^ spectrum of metabolite **M6** (hydroxylation) was analyzed and similar to metabolite **M5a** this compound exhibited a product ion (*m*/*z* 152.0163), indicating hydroxylation on the aniline ring of the molecule. Summarizing the results for SLU‐PP‐915, a total of seven metabolites, all of which are assigned to phase‐I transformations, were identified. An overview of the metabolic pattern of SLU‐PP‐915 is shown in Figure 9.

Within this study, MS^2^ experiments were conducted to yield a deeper understanding on the possible position of introduced functionalities during in vitro metabolism. However, using such experiments is oftentimes insufficient to determine the exact position of the metabolic transformations rather than their approximate location. To address this issue, the suspected metabolite has to be synthesized to confirm its structure, as also exemplified in this work. Additionally, all transformations described in this paper are observed using an in vitro metabolic approach. However, while this approach provides an overview of possible metabolites, it might not fully reflect the authentic human metabolic pattern and, thereby, plasma or urinary metabolic profiles. More complex metabolism studies, such as organ‐on‐a‐chip models or clinical trials, are required to gain comprehensive knowledge of the metabolism of pan‐ERR agonists.

## 4 Conclusion

The investigation of novel substances that might be misused as doping agents is one of the most crucial tasks in preventive doping research. Within this work, the analytical characterization of the drug candidates SLU‐PP‐332 and SLU‐PP‐915, two compounds that are of particular interest for doping control purposes, was accomplished. The obtained results may help implementing these substances into existing doping control methods. In addition, both substances were metabolized in vitro, and an in‐depth structural elucidation was presented. Moreover, in the case of SLU‐PP‐915, a total of three metabolites were synthesized in order to confirm their proposed structures and to provide reference material. Gathering the results of this work, an important foundation has been laid for enabling the detection of the investigated pan‐ERR agonists and their (potential) metabolites in doping control samples. Further research is needed to address the metabolic behavior in vivo and to ensure effective detection of both substances in human urine samples.

## Author Contributions

**Mario Thevis:** conceptualization, funding acquisition, writing – review and editing, methodology, supervision, project administration. **Tristan Möller**: conceptualization, conducted experiments, writing – original draft. **Oliver Krug:** conducted experiments.

## Funding

This project was conducted with support of the Manfred‐Donike Institute for Doping Analysis (Cologne, Germany), the Ingeborg‐Gross‐Foundation, and the Federal Chancellery of the Federal Republic of Germany (Berlin, Germany).

## Conflicts of Interest

The authors declare no conflicts of interest.

## Supporting information


**Figure S1:** ^1^H‐NMR of 2a
**Figure S2:** 13C‐APT NMR of 2a
**Figure S3:** 1H‐NMR of 2b
**Figure S4:** 13C‐APT NMR of 2
**Figure S5:** 1H‐NMR of SLU‐PP‐915
**Figure S6:** 13C‐APT NMR of SLU‐PP‐915
**Figure S7:** 1H‐NMR of SLU‐PP‐915‐Cl
**Figure S8:** 13C‐APT NMR of SLU‐PP‐915‐Cl
**Figure S9:** 1H‐NMR of M1
**Figure S10:** 13C‐APT NMR of M1
**Figure S11:** 1H‐NMR of M3
**Figure S12:** 13C‐APT NMR of M3
**Figure S13:** 13C‐APT NMR of M4
**Figure S14:** 1H‐NMR of M4

## Data Availability

Data supporting this study are available upon reasonable request from the corresponding author (M.T.).
