# Supplementary material for: In Vitro Metabolism and Analytical Characterization of SLU‐PP‐332 and SLU‐PP‐915: Novel Pan‐ERR Agonists With Doping Potential
Source: Rapid Commun Mass Spectrom. 2026 Jan 26;40(8):e70039. doi: 10.1002/rcm.70039 (PMC12835572; doi:10.1002/rcm.70039)

Supporting information

# *In‑Vitro* Metabolism and Analytical Characterization of SLU‑PP‑332 and SLU‑PP‑915: Novel Pan-ERR Agonists with Doping Potential

Tristan Möller^[1]^, Oliver Krug ^[1,2]^, Mario Thevis ^[1,2]^

[1] Center for Preventive Doping Research/Institute of Biochemistry, German Sport University Cologne

[2] European Monitoring Center for Emerging Doping Agents (EuMoCEDA)

[Fig. 1 ^1^H-NMR of 2a 2](file:///\\ad.mdiev.de\IT\Userdata\Ordnerumleitung\t.moeller\Documents\pan-ERR%20agonists\paper\Supporting%20information.docx#_Toc216265122)

[Fig. 2 ^13^C-APT NMR of 2a 2](file:///\\ad.mdiev.de\IT\Userdata\Ordnerumleitung\t.moeller\Documents\pan-ERR%20agonists\paper\Supporting%20information.docx#_Toc216265123)

[Fig. 3 ^1^H-NMR of 2b 3](file:///\\ad.mdiev.de\IT\Userdata\Ordnerumleitung\t.moeller\Documents\pan-ERR%20agonists\paper\Supporting%20information.docx#_Toc216265124)

[Fig. 4 ^13^C-APT NMR of 2 3](file:///\\ad.mdiev.de\IT\Userdata\Ordnerumleitung\t.moeller\Documents\pan-ERR%20agonists\paper\Supporting%20information.docx#_Toc216265125)

[Fig. 5 ^1^H-NMR of SLU-PP-915 4](file:///\\ad.mdiev.de\IT\Userdata\Ordnerumleitung\t.moeller\Documents\pan-ERR%20agonists\paper\Supporting%20information.docx#_Toc216265126)

[Fig. 6 ^13^C-APT NMR of SLU-PP-915 4](file:///\\ad.mdiev.de\IT\Userdata\Ordnerumleitung\t.moeller\Documents\pan-ERR%20agonists\paper\Supporting%20information.docx#_Toc216265127)

[Fig. 7 ^1^H-NMR of SLU-PP-915-Cl 5](file:///\\ad.mdiev.de\IT\Userdata\Ordnerumleitung\t.moeller\Documents\pan-ERR%20agonists\paper\Supporting%20information.docx#_Toc216265128)

[Fig. 8 ^13^C-APT NMR of SLU-PP-915-Cl 5](file:///\\ad.mdiev.de\IT\Userdata\Ordnerumleitung\t.moeller\Documents\pan-ERR%20agonists\paper\Supporting%20information.docx#_Toc216265129)

[Fig. 9 ^1^H-NMR of M1 6](file:///\\ad.mdiev.de\IT\Userdata\Ordnerumleitung\t.moeller\Documents\pan-ERR%20agonists\paper\Supporting%20information.docx#_Toc216265130)

[Fig. 10 ^13^C-APT NMR of M1 6](file:///\\ad.mdiev.de\IT\Userdata\Ordnerumleitung\t.moeller\Documents\pan-ERR%20agonists\paper\Supporting%20information.docx#_Toc216265131)

[Fig. 11 ^1^H-NMR of M3 7](file:///\\ad.mdiev.de\IT\Userdata\Ordnerumleitung\t.moeller\Documents\pan-ERR%20agonists\paper\Supporting%20information.docx#_Toc216265132)

[Fig. 12 ^13^C-APT NMR of M3 7](file:///\\ad.mdiev.de\IT\Userdata\Ordnerumleitung\t.moeller\Documents\pan-ERR%20agonists\paper\Supporting%20information.docx#_Toc216265133)

[Fig. 13 ^13^C-APT NMR of M4 8](file:///\\ad.mdiev.de\IT\Userdata\Ordnerumleitung\t.moeller\Documents\pan-ERR%20agonists\paper\Supporting%20information.docx#_Toc216265134)

[Fig. 14 ^1^H-NMR of M4 8](file:///\\ad.mdiev.de\IT\Userdata\Ordnerumleitung\t.moeller\Documents\pan-ERR%20agonists\paper\Supporting%20information.docx#_Toc216265135)

Fig. 1 ^1^H-NMR of 2a


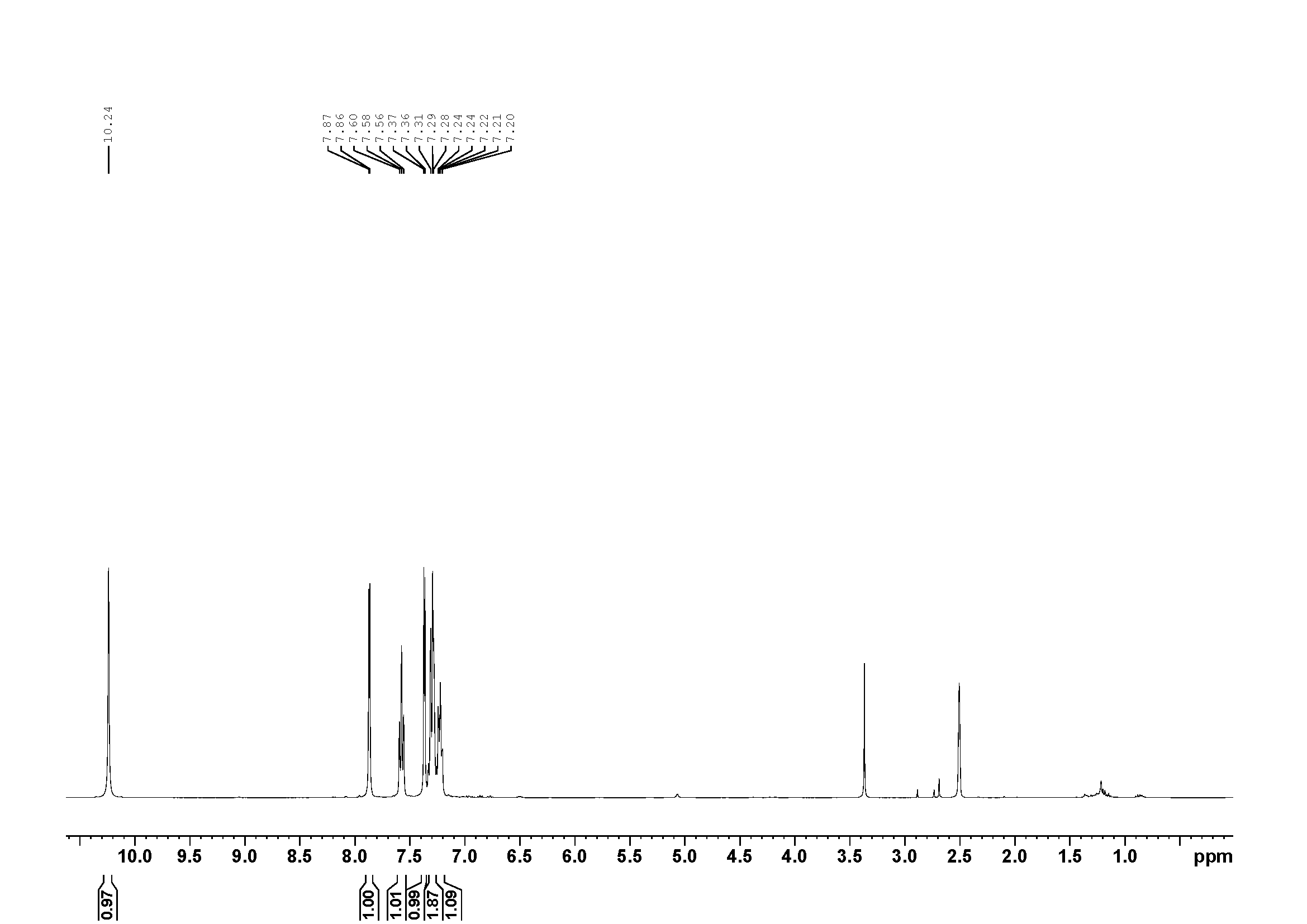

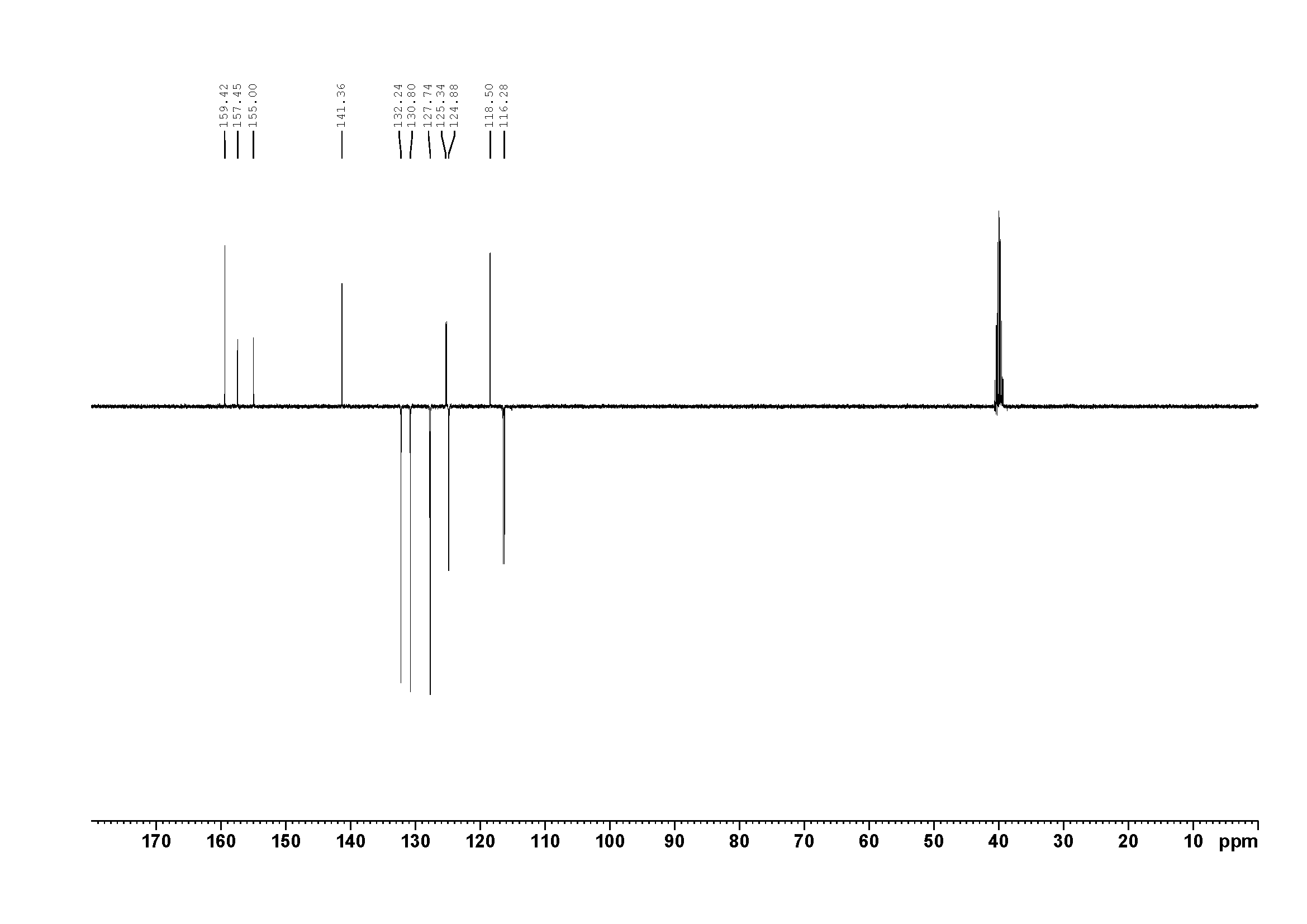


Fig. 2 ^13^C-APT NMR of 2a

Fig. 3 ^1^H-NMR of 2b


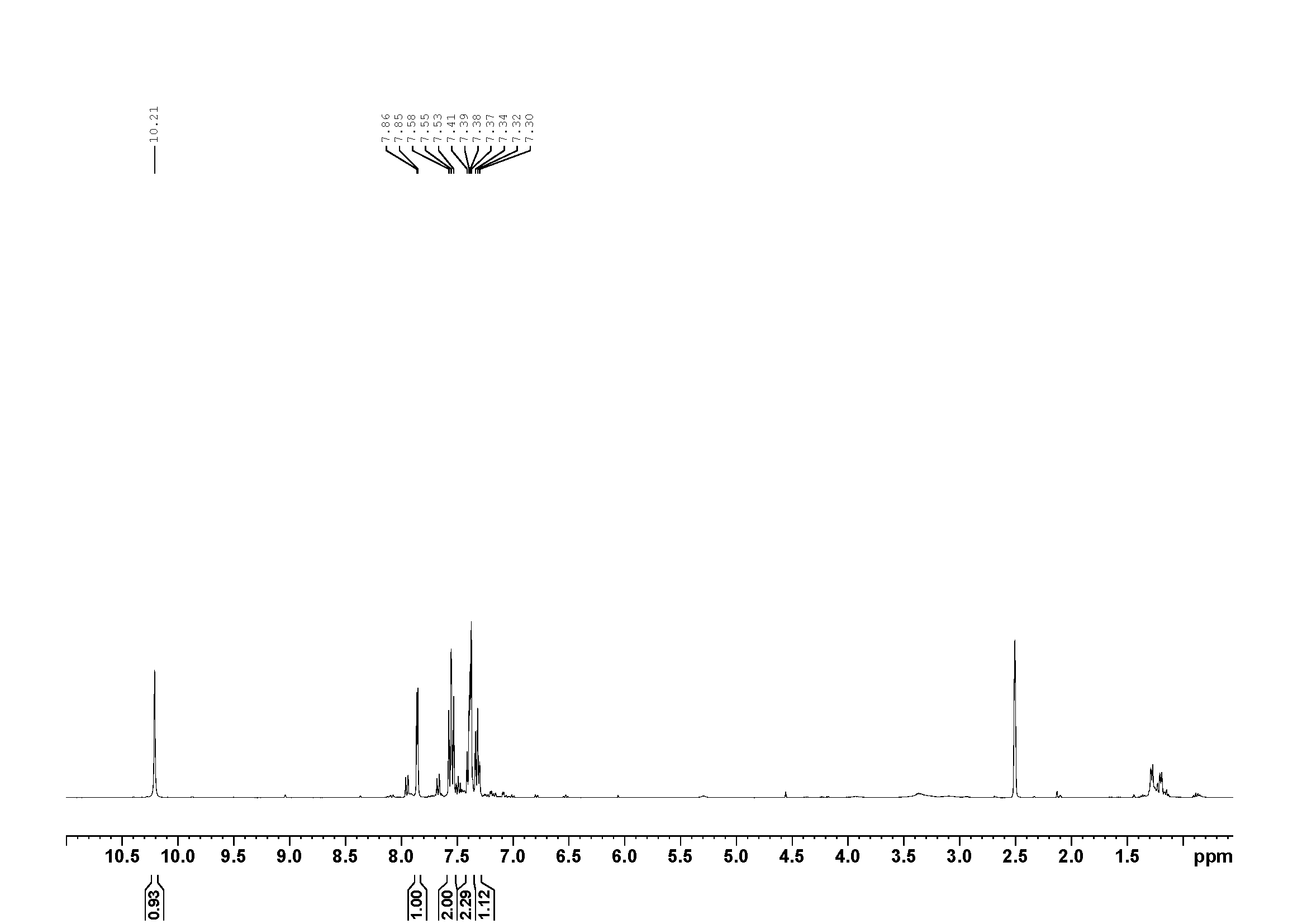


Fig. 4 ^13^C-APT NMR of 2


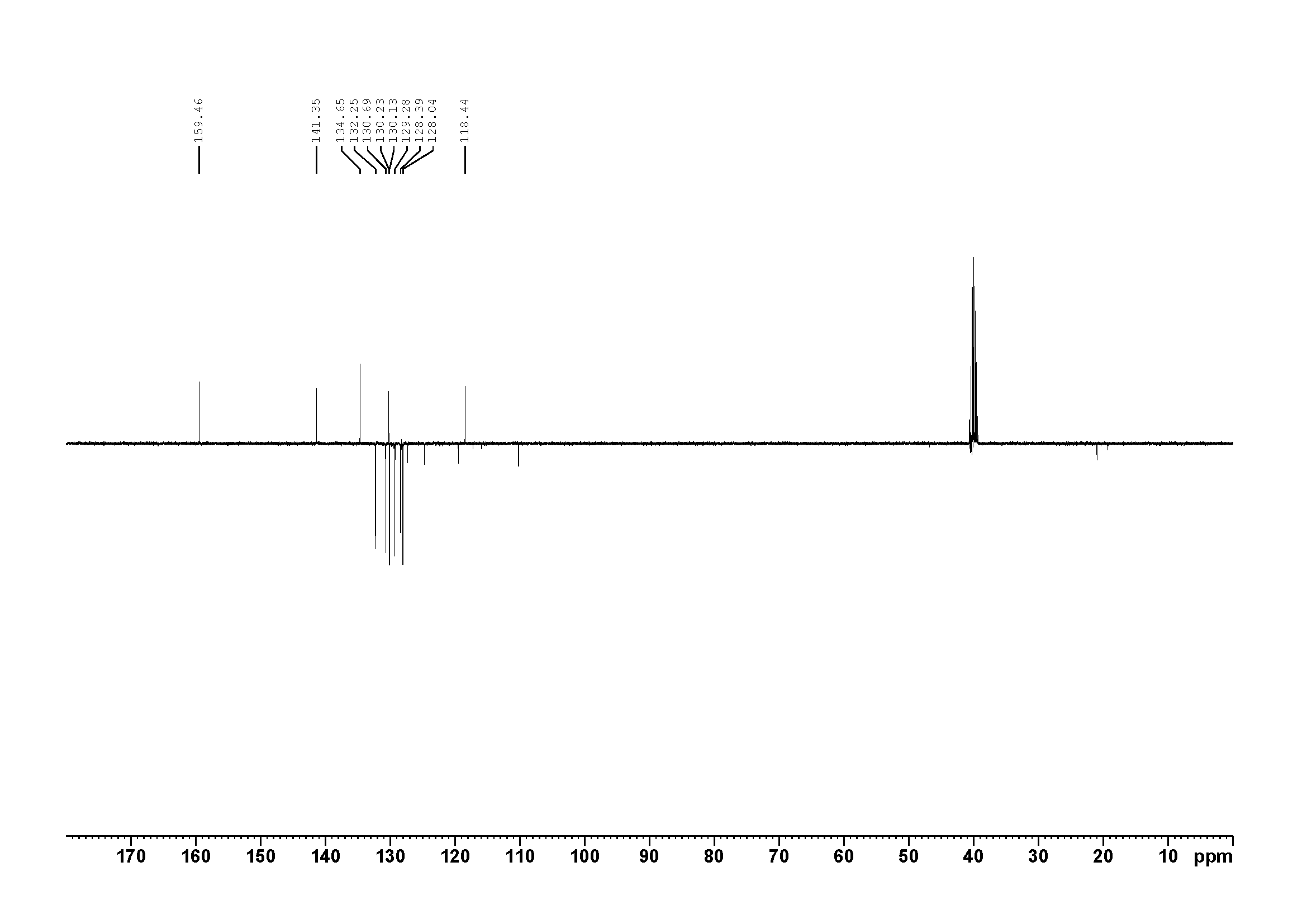


Fig. 5 ^1^H-NMR of SLU-PP-915


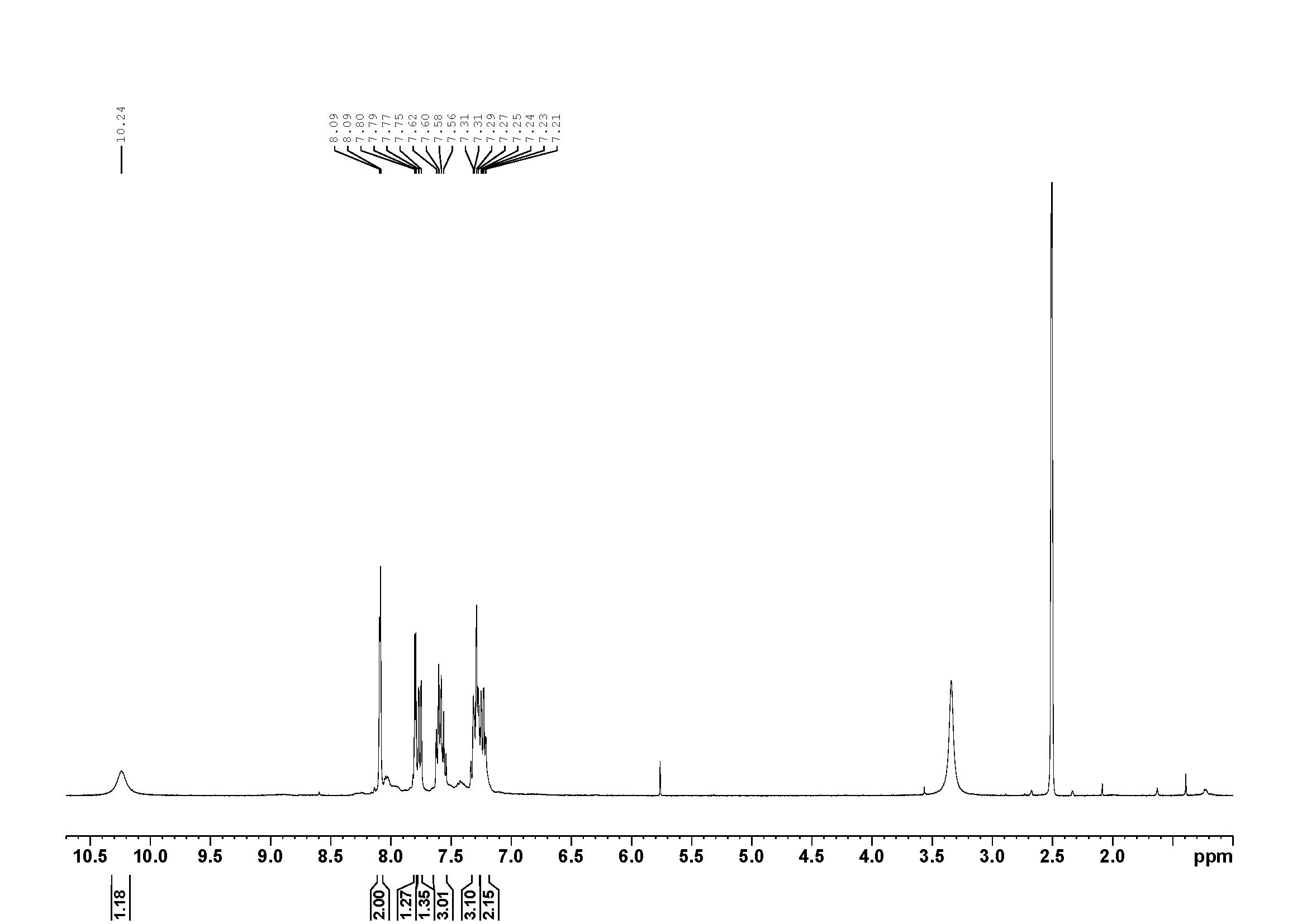


Fig. 6 ^13^C-APT NMR of SLU-PP-915


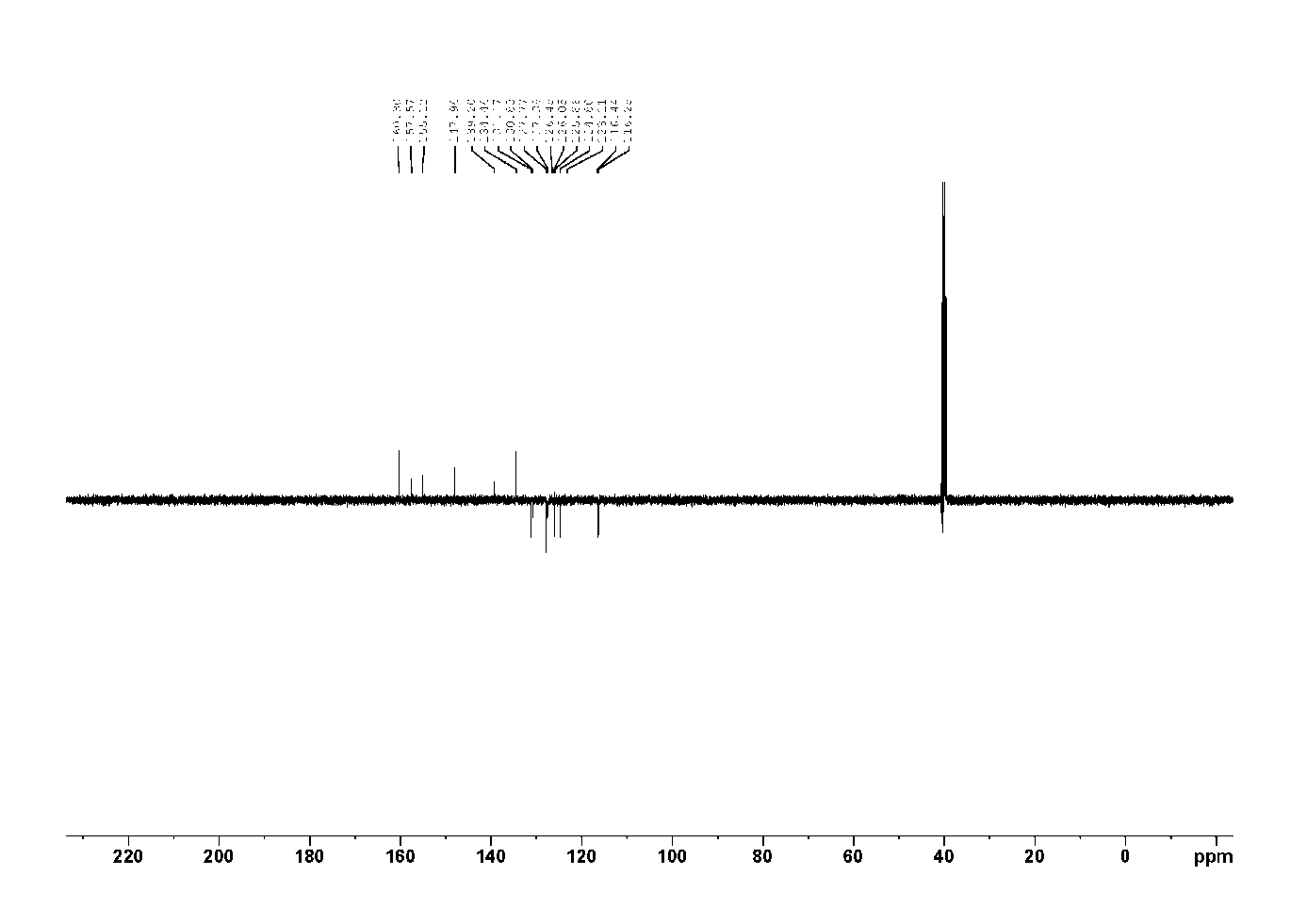


Fig. 7 ^1^H-NMR of SLU-PP-915-Cl


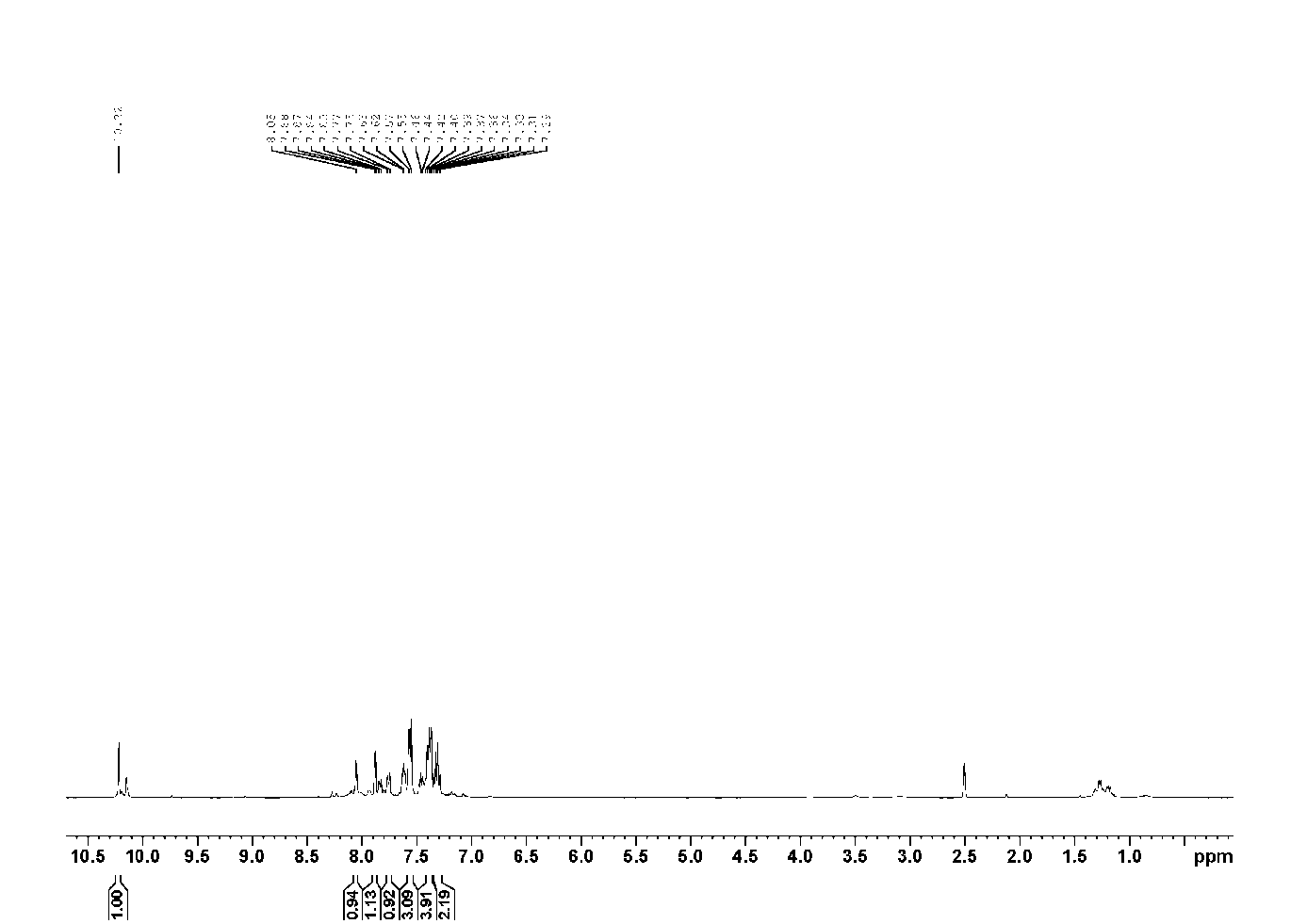


Fig. 8 ^13^C-APT NMR of SLU-PP-915-Cl


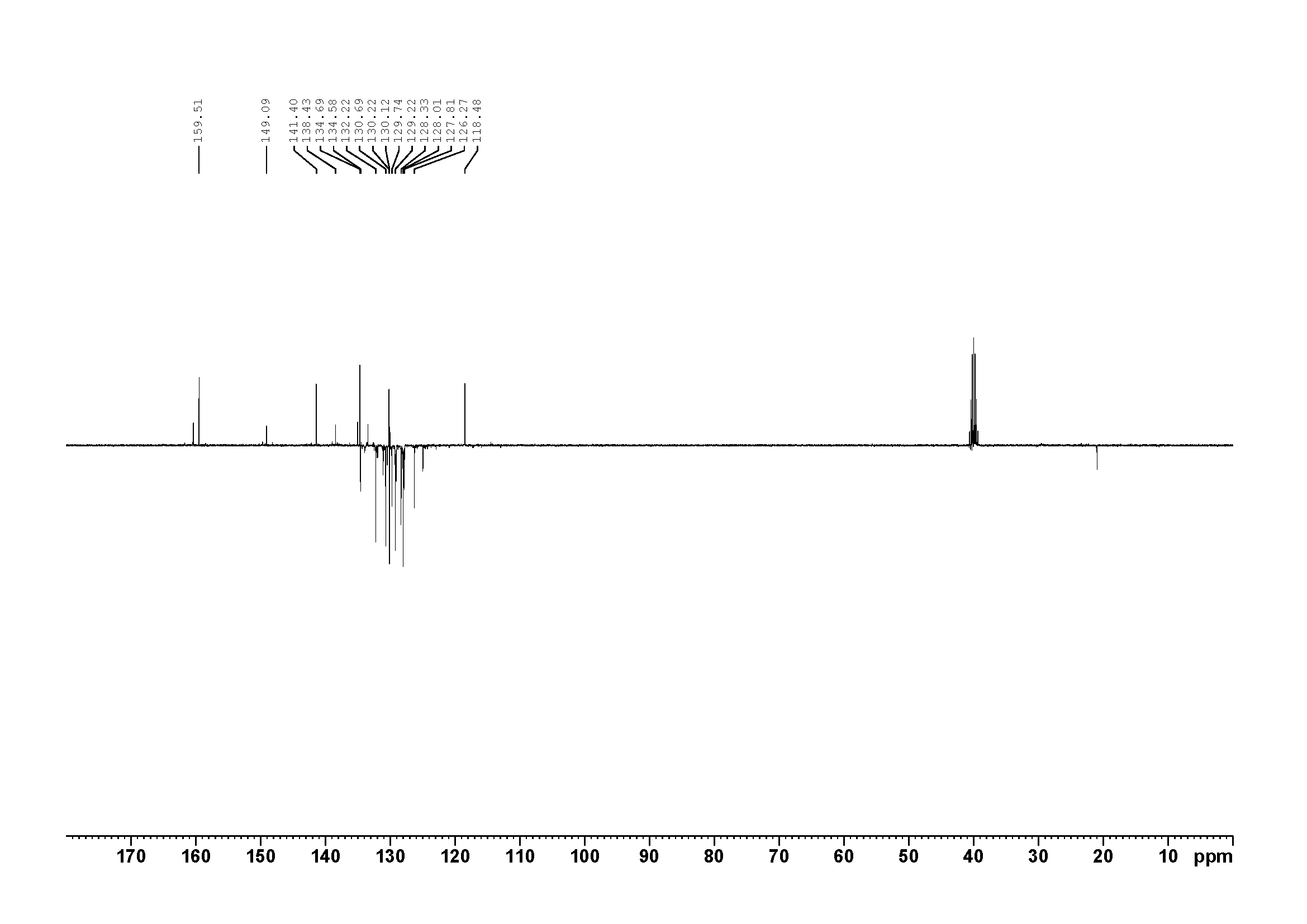


Fig. 9 ^1^H-NMR of M1


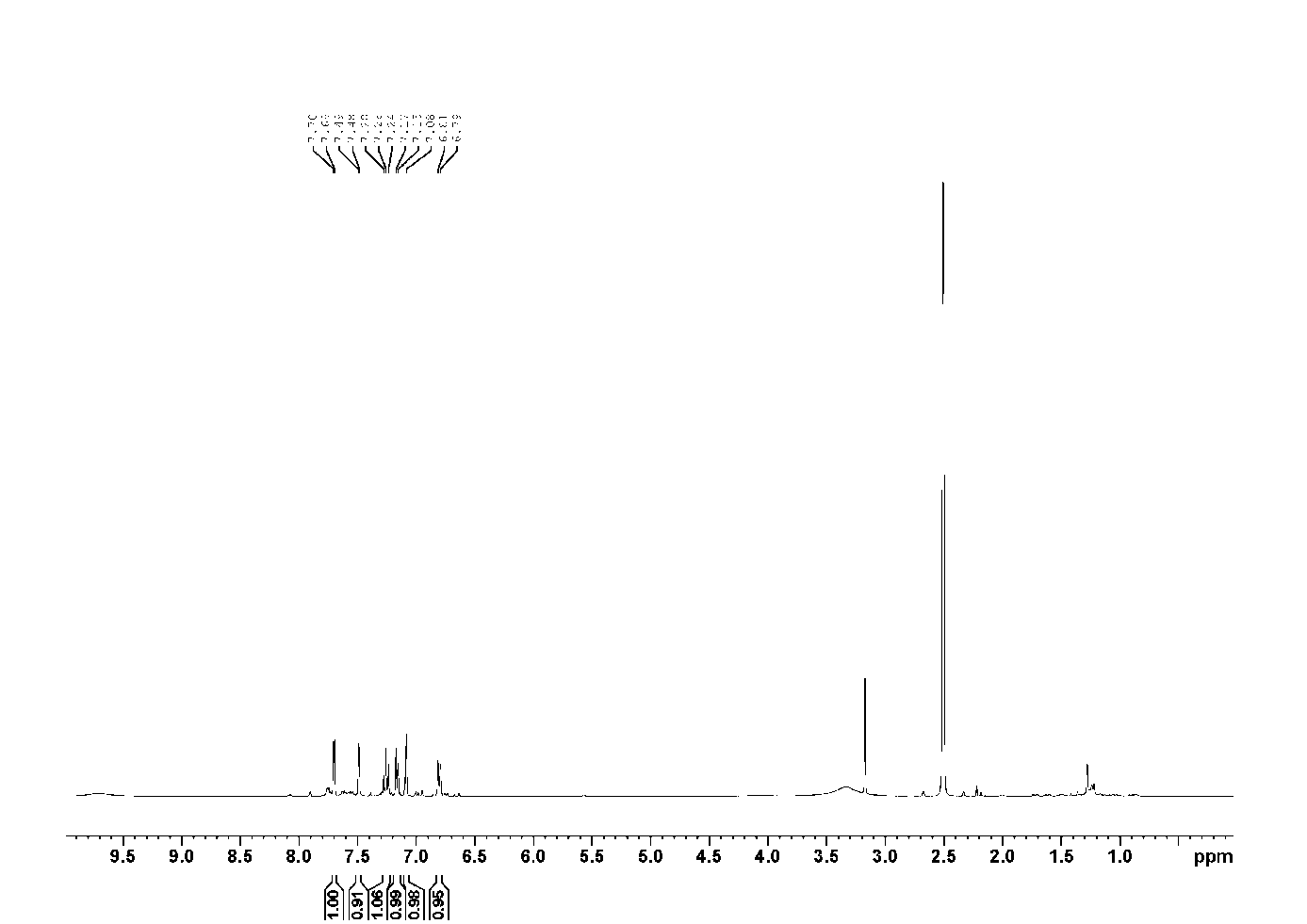


Fig. 10 ^13^C-APT NMR of M1


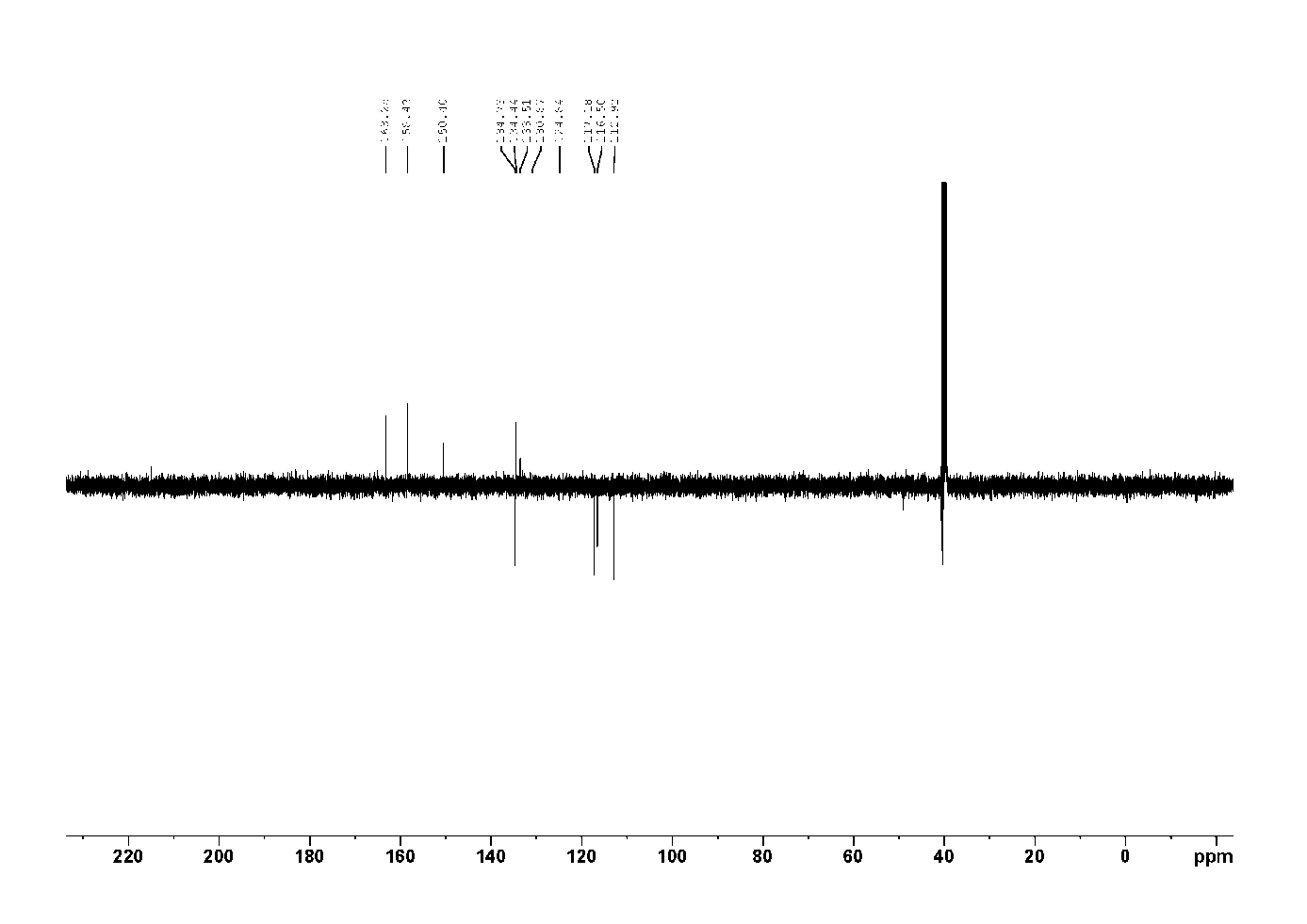


Fig. 11 ^1^H-NMR of M3


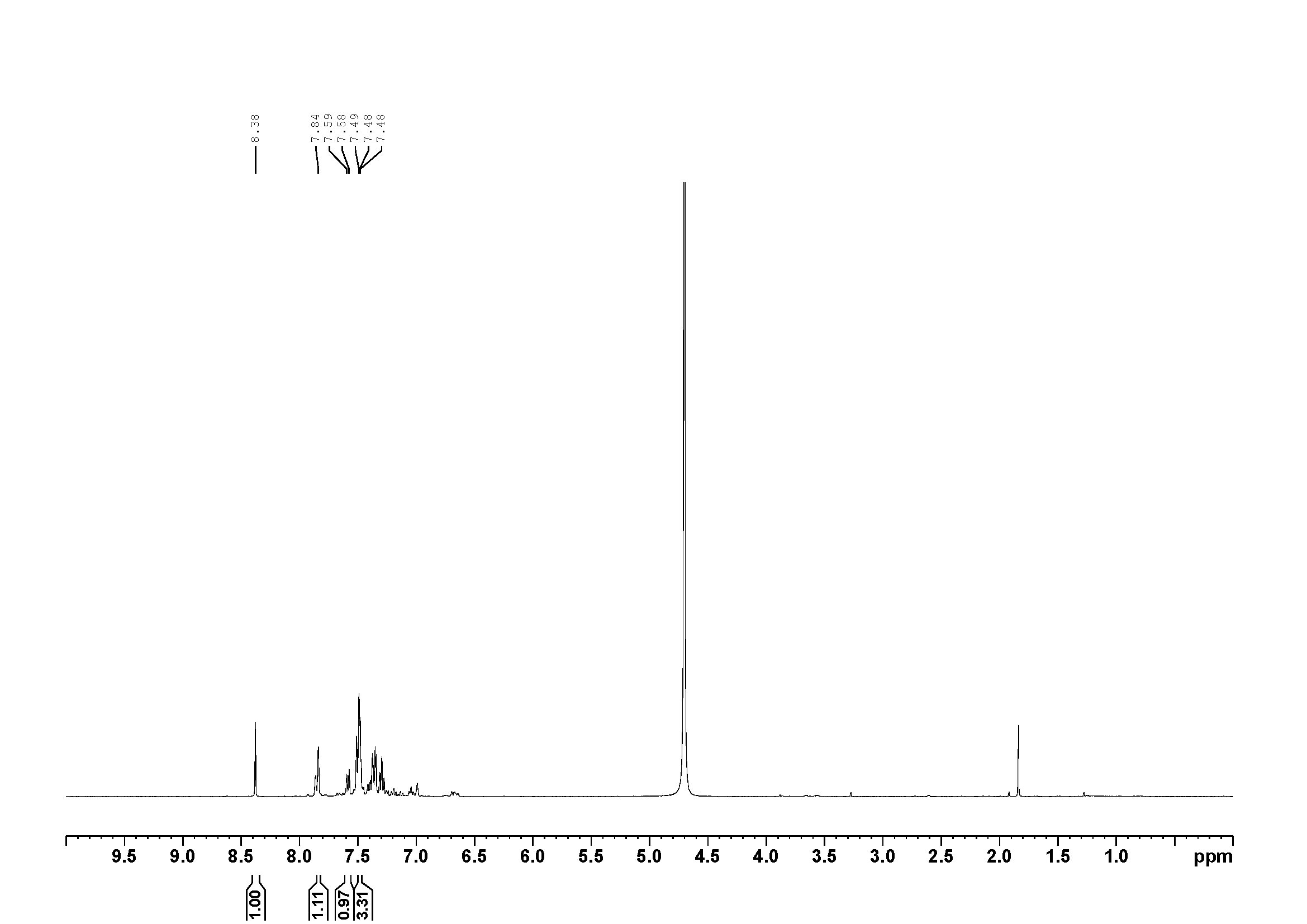


Fig. 12 ^13^C-APT NMR of M3


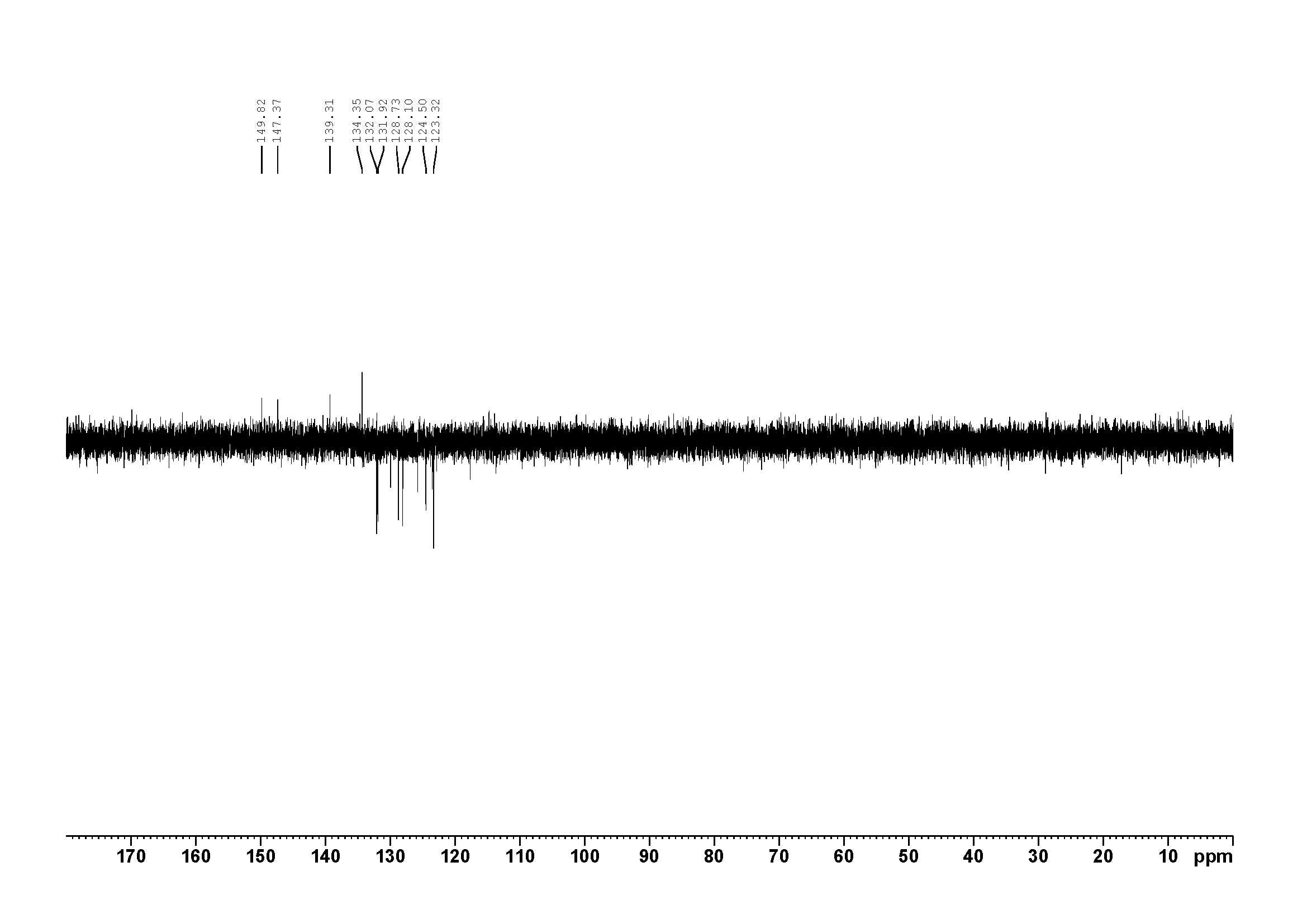

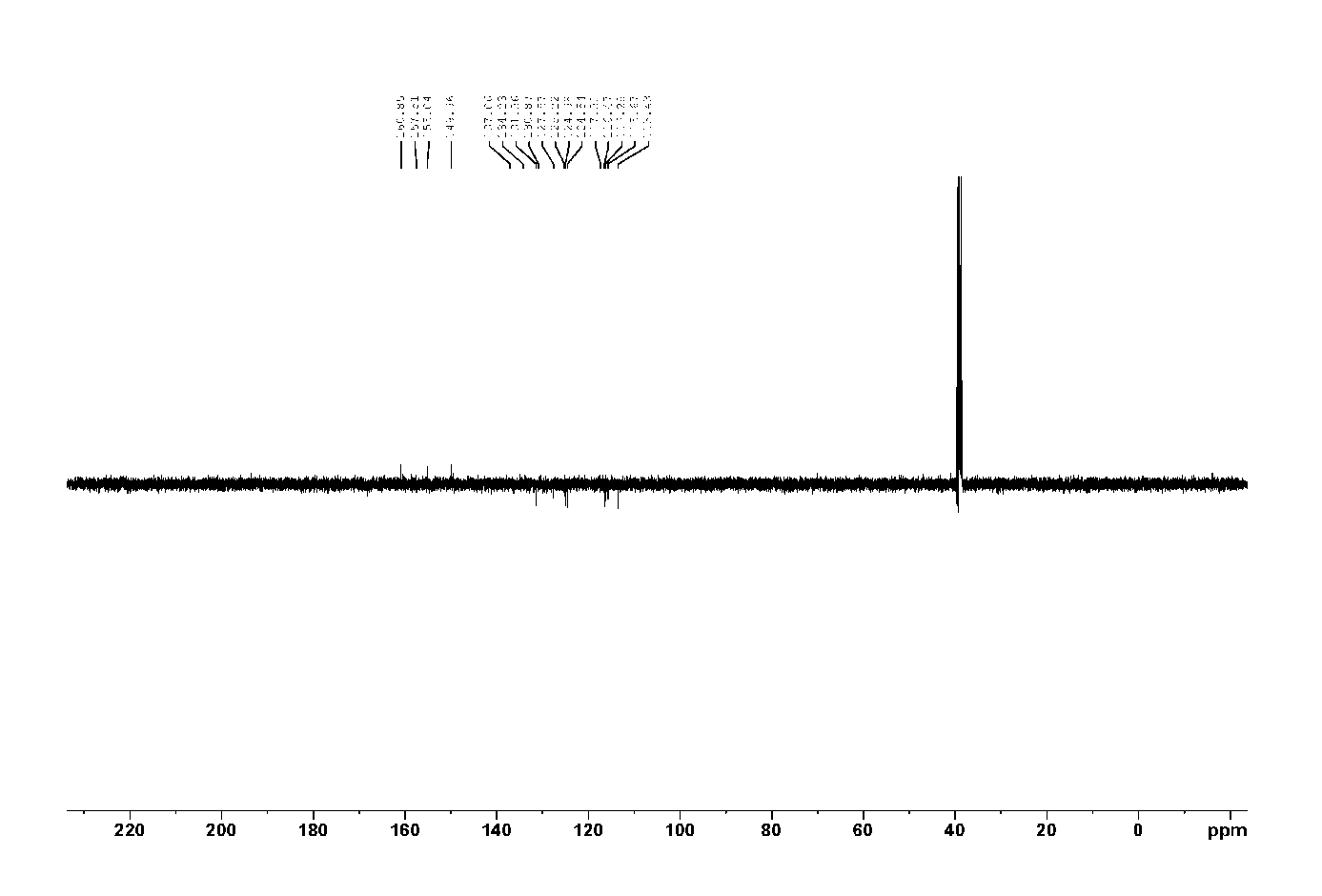


Fig. 13 ^13^C-APT NMR of M4

Fig. 14 ^1^H-NMR of M4


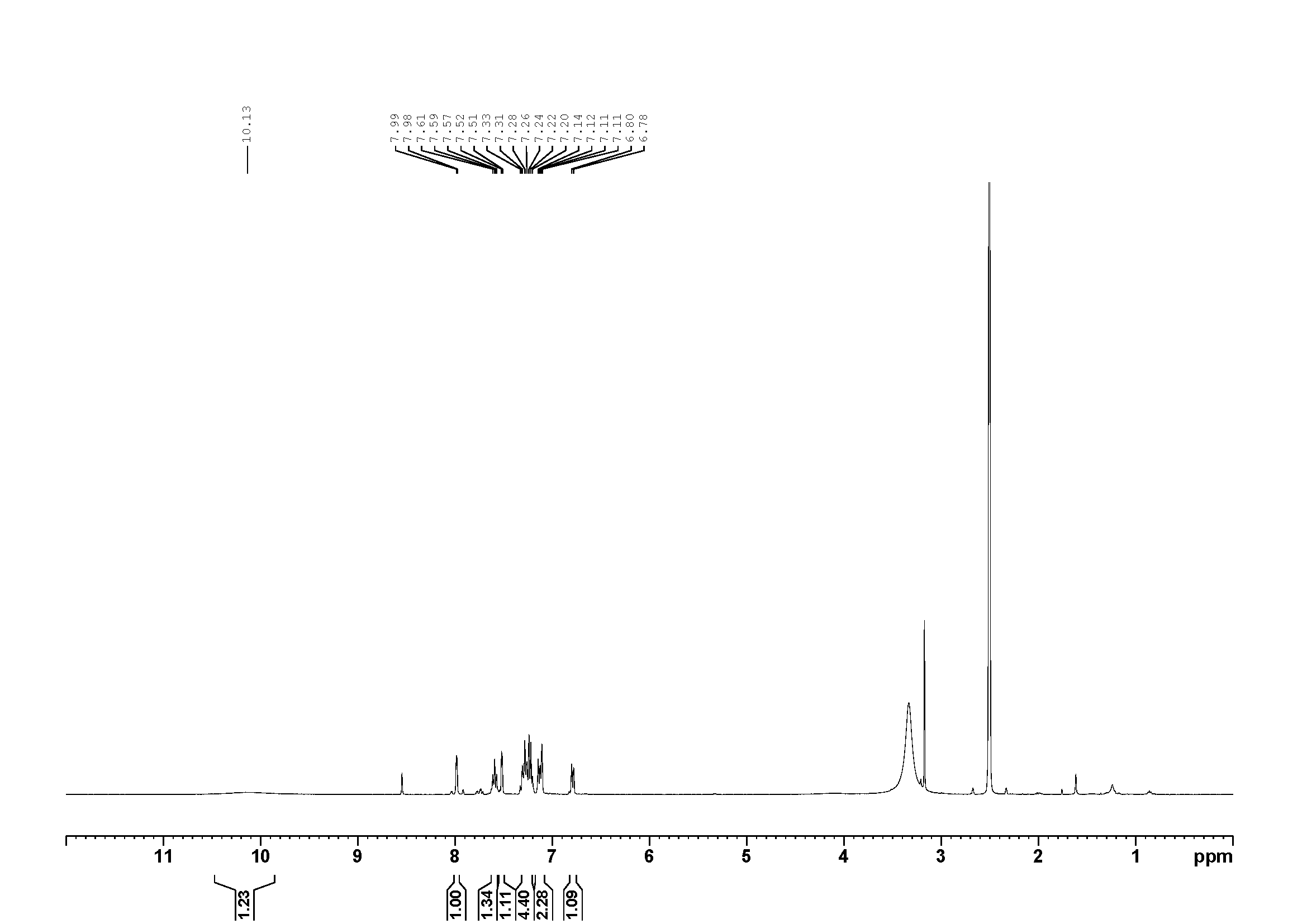

Supplement: Supplementary file 1 — Figure S1: 1H‐NMR of 2a Figure S2: 13C‐APT NMR of 2a Figure S3: 1H‐NMR of 2b Figure S4: 13C‐APT NMR of 2 Figure S5: 1H‐NMR of SLU‐PP‐915 Figure S6: 13C‐APT NMR of SLU‐PP‐915 Figure S7: 1H‐NMR of SLU‐PP‐915‐Cl Figure S8: 13C‐APT NMR of SLU‐PP‐915‐Cl Figure S9: 1H‐NMR of M1 Figure S10: 13C‐APT NMR of M1 Figure S11: 1H‐NMR of M3 Figure S12: 13C‐APT NMR of M3 Figure S13: 13C‐APT NMR of M4 Figure S14: 1H‐NMR of M4 [file RCM-40-e70039-s001.docx]
